# Global shortfalls in documented actions to conserve biodiversity

**DOI:** 10.1038/s41586-024-07498-7

**Published:** 2024-06-05

**Authors:** Rebecca A. Senior, Ruby Bagwyn, Danyan Leng, Alexander K. Killion, Walter Jetz, David S. Wilcove

**Affiliations:** 1 Princeton School of Public and International Affairs, Princeton University, Princeton, NJ USA; 2 Conservation Ecology Group, Department of Biosciences, Durham University, Durham, UK; 3 Williams College, Williamstown, MA USA; 4 Department of Ecology and Evolutionary Biology, Yale University, New Haven, CT USA; 5 Center for Biodiversity and Global Change, Yale University, New Haven, CT USA; 6 Department of Ecology and Evolutionary Biology, Princeton University, Princeton, NJ USA

**Keywords:** Biodiversity, Conservation biology, Conservation biology

## Abstract

Threatened species are by definition species that are in need of assistance. In the absence of suitable conservation interventions, they are likely to disappear soon^1^. There is limited understanding of how and where conservation interventions are applied globally, or how well they work^2,3^. Here, using information from the International Union for Conservation of Nature Red List and other global databases, we find that for species at risk from three of the biggest drivers of biodiversity loss—habitat loss, overexploitation for international trade and invasive species^4^—many appear to lack the appropriate types of conservation interventions. Indeed, although there has been substantial recent expansion of the protected area network, we still find that 91% of threatened species have insufficient representation of their habitats within protected areas. Conservation interventions are not implemented uniformly across different taxa and regions and, even when present, have infrequently led to substantial improvements in the status of species. For 58% of the world’s threatened terrestrial species, we find conservation interventions to be notably insufficient or absent. We cannot determine whether such species are truly neglected, or whether efforts to recover them are not included in major conservation databases. If they are indeed neglected, the outlook for many of the world’s threatened species is grim without more and better targeted action.

## Abstract (web summary)

A global assessment of conservation interventions for threatened species across a range of data sources finds that interventions for most species remain insufficient or absent.

## Main

The need for greater attention to biodiversity conservation is unequivocal and urgent^5^. The world is entering its sixth mass extinction event^6^, the first that is attributable to a single species: *Homo sapiens*. Biodiversity loss is a global concern and the focus of multiple international commitments, including those recently pledged in the Kunming–Montreal Global Biodiversity Framework of the United Nations Convention on Biological Diversity. Nevertheless, species extinctions continue to accumulate^7^.

Conservation efforts can forestall species extinctions^8,9^, but funding remains insufficient^10,11^. Moreover, if effort is poorly targeted relative to risk^12^, fewer species will be saved than is otherwise possible. Prospects for biodiversity can be improved through increased resources and more efficient allocation of the scarce resources that are available. More efficient allocation requires that we identify the conservation interventions that decrease species’ risks of extinction^2^, along with the interventions that have been implemented and where they have been implemented.

Until recently, little attention has been given to assessing what works in conservation^2,13^. Assessments of the effectiveness of protected areas (PAs) are a notable exception, with multiple studies finding that well-managed PAs mitigate biodiversity loss^14,15^. Similarly, extensive data demonstrate the benefit of invasive species eradication efforts for island biotas^16^. There are, however, many other types of conservation interventions^17^, many of which have yet to be assessed together and at scale.

The International Union for the Conservation of Nature (IUCN) conducts species-level assessments of extinction risk via its Red List of Threatened Species^1,18^. Assessors also compile information on the conservation interventions implemented for each species. Studies have used these data to identify interventions associated with decreased extinction risk in birds and mammals^8,19,20^. Here we provide a global assessment of patterns of conservation action across regions and taxonomic groups by supplementing the Red List data with a manual review of their ‘Conservation Actions in Place’ text, combined with four other databases on specific interventions: Map of Life^21^ (https://mol.org); the World Database on Protected Areas^22^ (WDPA); Species+ (https://speciesplus.net); and the Database of Island Invasive Species Eradications^23^ (DIISE). We consider all 5,963 terrestrial threatened species (those classed as Vulnerable, Endangered or Critically Endangered) within taxonomic families that have been comprehensively assessed for the Red List (Supplementary Table 1).

We initially focus on three of the greatest threats to biodiversity: habitat loss (including habitat degradation), overexploitation for international trade and invasive species^4^. Each has a clearly matched conservation intervention: habitat protection (via PAs), trade control and invasive species control, respectively. Although we recognize the potentially severe impact of domestic overexploitation^24^, we focus on international trade only, given the greater availability of data on this threat and its matched intervention.

Regarding habitat loss, we consider meaningful habitat protection to be in place only if a species-specific representation threshold of overlap is met, as defined by the Species Protection Score of Map of Life (Methods). The Species Protection Score contributes to the Species Protection Index, an indicator of the United Nations Global Biodiversity Framework that addresses species representation^21,25^. Even where sufficient representation within PAs is achieved, we note that species may still be threatened by habitat loss where PAs are poorly managed or subject to downgrading, downsizing or degazettement^26^. We also acknowledge the value of interventions besides PAs, including other effective area-based conservation measures^27^, spatial planning^28^ and habitat restoration. However, we lack comparably comprehensive data to include them here.

For species threatened by any of the three major drivers of biodiversity loss outlined above, we begin with two key questions: (1) what proportion of species receive the appropriate type(s) of conservation intervention? and (2) does a species’ taxonomy, biogeography or extinction risk influence the likelihood that the appropriate intervention will be made? Finally, we consider all categories of in situ conservation interventions documented by the Red List^17^—additionally including reintroduction, international legislation and education—to identify threatened species that have no documented conservation interventions. For species that lack documented conservation interventions, we explore whether the apparent lack of conservation attention is qualitatively associated with changes in the species status on the Red List.

**Fig. 1:**
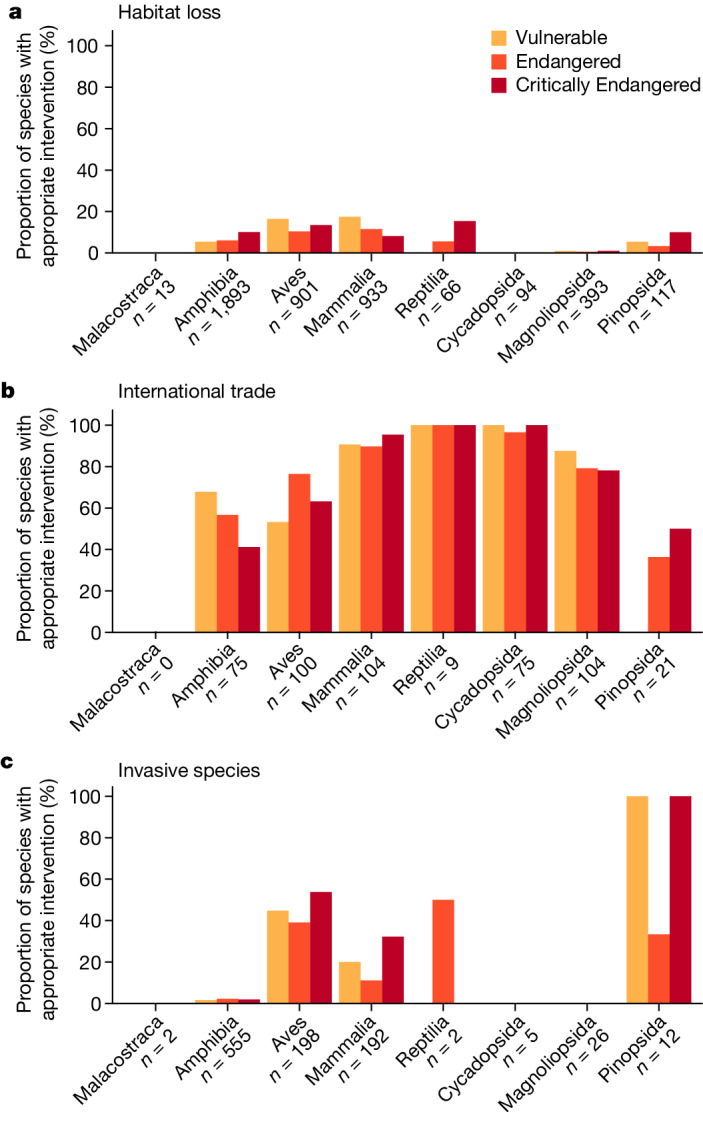
The proportion of threatened species documented as receiving the appropriate type of conservation intervention to tackle three major threats to biodiversity. **a**–**c**, The threats are habitat loss (**a**), overexploitation for international trade (**b**), and invasive species (**c**). Bar colours denote Red List categories. *n* represents the total number of species included in our analyses, by taxonomic class and threat.

Globally, we find substantial shortcomings in documented conservation interventions for the world’s threatened terrestrial species. Most threatened species at risk of overexploitation for international trade are documented as being subject to international trade control (76%). However, of those under threat from habitat loss, only 9% have sufficient representation of their habitat in PAs to meet target thresholds. As noted elsewhere, small-ranged species are particularly poorly represented in the current PA network^26,29,30^. Among species threatened primarily by invasive species, only 15% are documented as receiving invasive species control (Fig. 1). If we relax the criterion for habitat protection to include mere occurrence in at least one PA, we find that 75% of species threatened by habitat loss are covered (Supplementary Figs. 2 and 3). Some taxonomic biases also exist in the extent to which species’ threats are documented as being appropriately addressed. For example, 0.76% and 0% of threatened flowering plants in the class Magnoliopsida (Fig. 1) are documented as receiving meaningful habitat protection or invasive species control, respectively (Fig. 1), whereas 14%, 63% and 46% of threatened birds are documented as receiving the appropriate interventions to tackle habitat loss, international trade and invasive species, respectively.

**Fig. 2:**
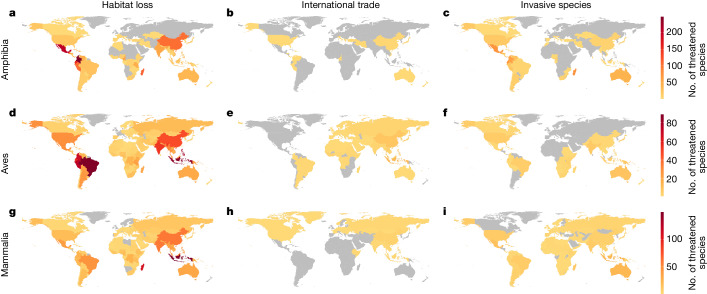
The total number of threatened species within each country apparently lacking the appropriate type of conservation intervention for three major threats. **a**–**i**, Results are summarized for each taxonomic class: Amphibia (**a**–**c**), Aves (**d**–**f**) and Mammalia (**g**–**i**); and for each of the three major threats to biodiversity with a clearly matched intervention: habitat loss (**a**,**d**,**g**), overexploitation for international trade (**b**,**e**,**h**) and invasive species (**c**,**f**,**i**). For clarity, we include here only the three vertebrate classes that have range data and have been comprehensively assessed; full results across all assessed families are presented in Extended Data Fig. 1.

**Extended Data Fig. 1:**
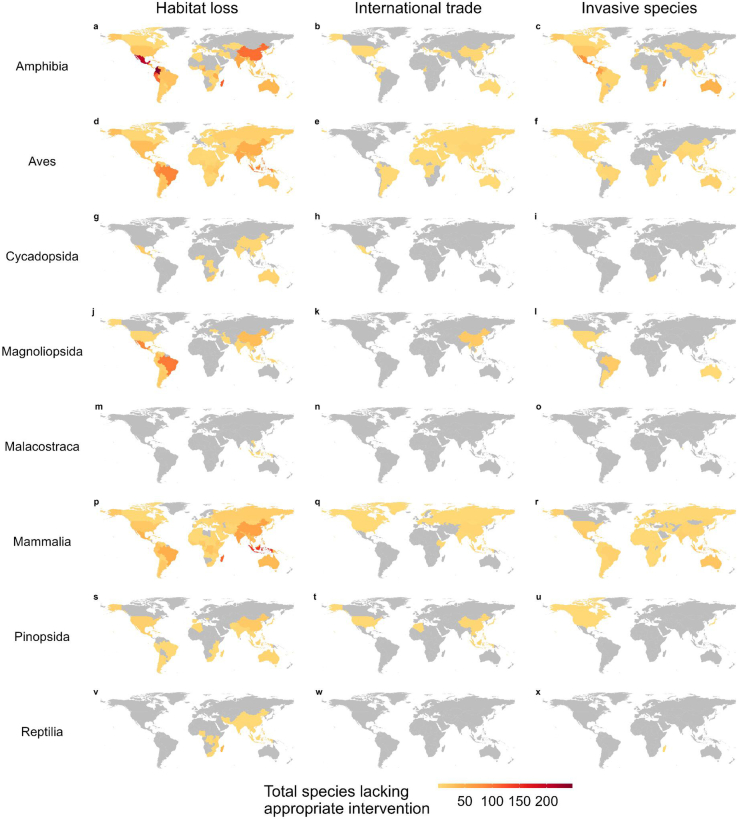
The total number of threatened species within each country lacking the appropriate conservation intervention. Results are summarised for each taxonomic group (Amphibia = a-c; Aves = d-f; Cycadopsida = g-i; Magnoliopsida = j-l; Malacostraca = m-o; Mammalia = p-r; Pinopsida = s-u; Reptilia = v-x) and for each of the three major threats to biodiversity with a clearly matched intervention (habitat loss = left column; overexploitation for international trade = middle column; invasive species = right column).

**Extended Data Fig. 2:**
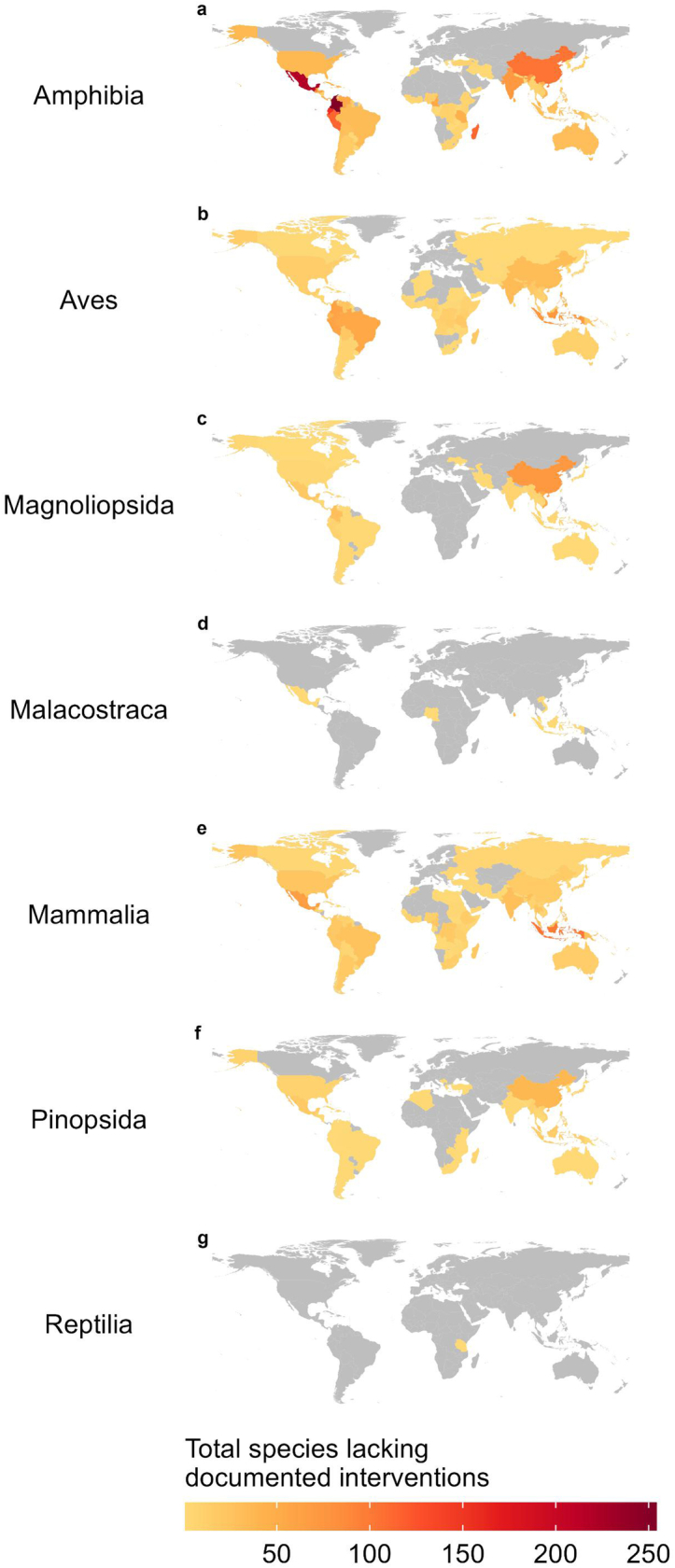
The total number of threatened species lacking any documented conservation interventions, in each country and taxonomic class. Taxa are: Amphibia, a; Aves, b; Magnoliopsida, c; Malacostraca, d; Mammalia, e; Pinopsida, f; Reptilia, g.

**Extended Data Fig. 3:**
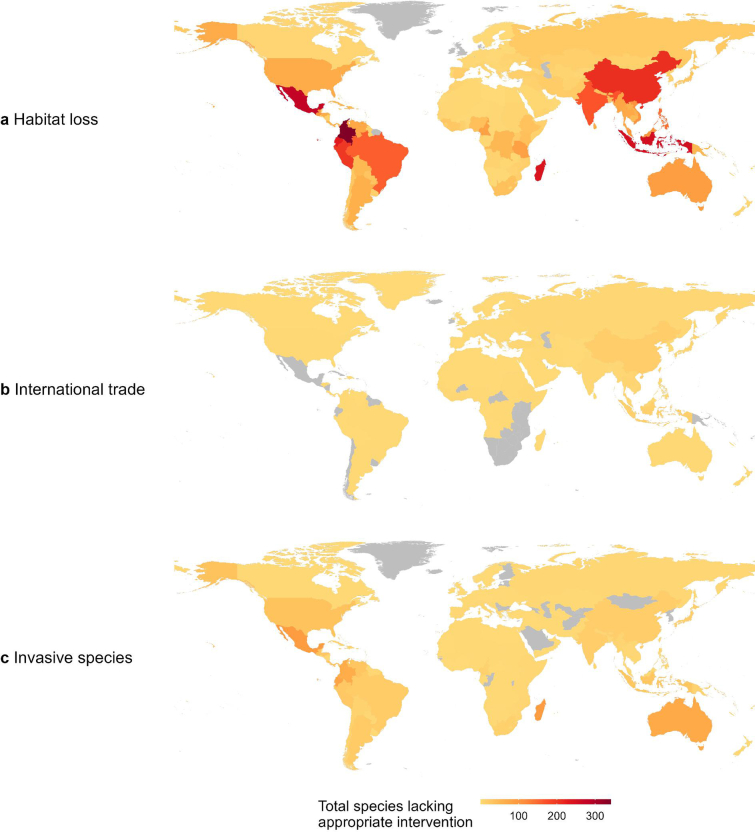
The total number of threatened species lacking any documented conservation intervention in each country, for each of the three major threats to biodiversity with a clearly matched intervention. Threats are: habitat loss, a; overexploitation for international trade, b; invasive species, c. Results are summed across the three vertebrate classes that have range data and have been comprehensively assessed: amphibians, birds and mammals (as in Fig. 2 in the main text).

**Extended Data Fig. 4:**
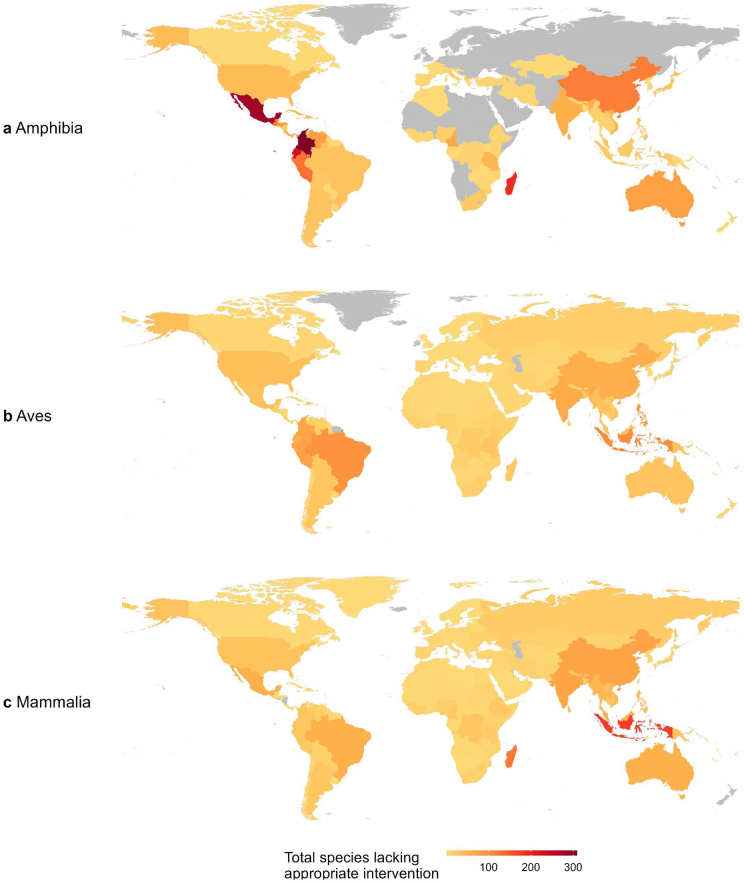
The total number of threatened species lacking any documented conservation intervention in each country, summed across the three major threats to biodiversity with a clearly matched intervention. As in Fig. 2 in the main text, we consider here only the three vertebrate classes that have range data and have been comprehensively assessed: Amphibia (a), Aves (b) and Mammalia (c). Note that the same species is counted multiple times if they face more than one of the three major threats.

The distribution of species lacking appropriate types of conservation intervention shows considerable geographic variability (*P* < 0.001) (Fig. 2 and Extended Data Figs. 1–4). Several regions contain high numbers of amphibians requiring invasive species control (*P* < 0.001), including Madagascar, Central America and Australia (Fig. 2c). The majority of cases (74%) in which control of invasive species is needed but lacking pertain to a lack of control of chytrid fungus, *Batrachochytrium dendrobatidis*, for which there is not yet an effective treatment^31^.

With respect to habitat loss, amphibians lack meaningful habitat protection in Central America (Fig. 2a) and mammals are notably lacking in habitat protection in Indonesia (Fig. 2g), as are birds in South America, Central America and Indonesia (Fig. 2d). Most species threatened by international trade are documented as receiving some international trade control (Fig. 1b), although relatively high numbers of exploited birds in Indonesia seemingly lack such protection (Fig. 2e and Extended Data Fig. 1).

**Fig. 3:**
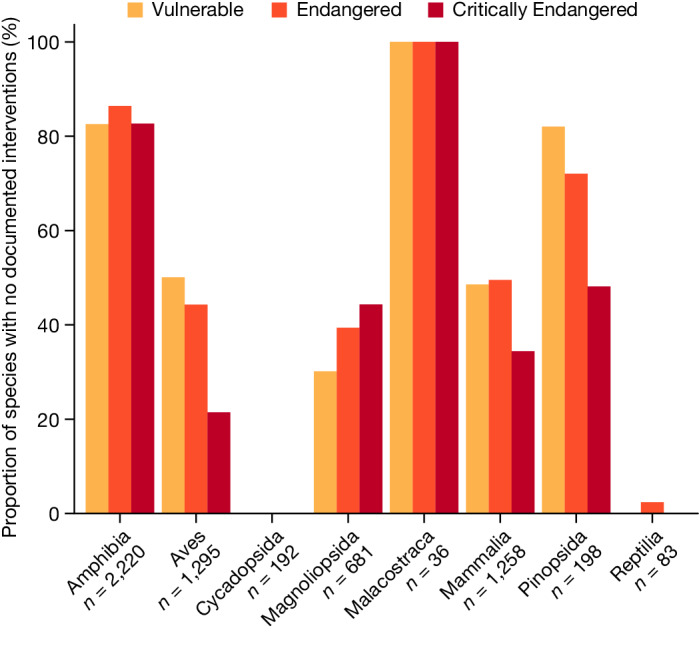
The proportion of threatened species lacking meaningful habitat protection or any of the other six categories of documented conservation interventions, irrespective of threat. Bar colours denote Red List categories: Vulnerable (yellow), Endangered (orange), and Critically Endangered (red). *n* represents the total number of species included in our analyses, by taxonomic class.

Finally, we assessed how many of the threatened species in our database are not documented as receiving meaningful habitat protection or any of the other categories of conservation intervention, expanded to include additional measures such as reintroduction^17^. Overall, we find that 58% (3,467 out of 5,963) of threatened terrestrial species lack meaningful habitat protection or any other documented conservation interventions (Fig. 3). We emphasize that this percentage is derived on the basis of Map of Life’s Species Protection Score (https://mol.org/indicators), which uses a species-specific representation threshold to determine whether a species has meaningful representation in PAs^21^. When we relax that threshold to whether a species occurs in any PA to any extent (as determined by the Red List), the proportion falls to 19% (1,105 out of 5,963; Supplementary Figs.  4 and 5). Taxa such as amphibians fare worse than others (deviance = 1,533, d.f. = 7, *P* < 0.001), with 84% lacking meaningful habitat protection or any other documented conservation interventions, compared with 44% for threatened birds. Taxonomic biases probably result from increased attention to charismatic and easily studied groups^12,32,33^, which also translates to more frequent Red List assessments. Across some (but not all) taxa, species at greatest risk of extinction are more likely to have documented attention (deviance = 19, d.f. = 1, *P* < 0.001). This corroborates Luther et al.^19^, who concluded that species at greater risk of extinction receive more conservation attention.

**Fig. 4:**
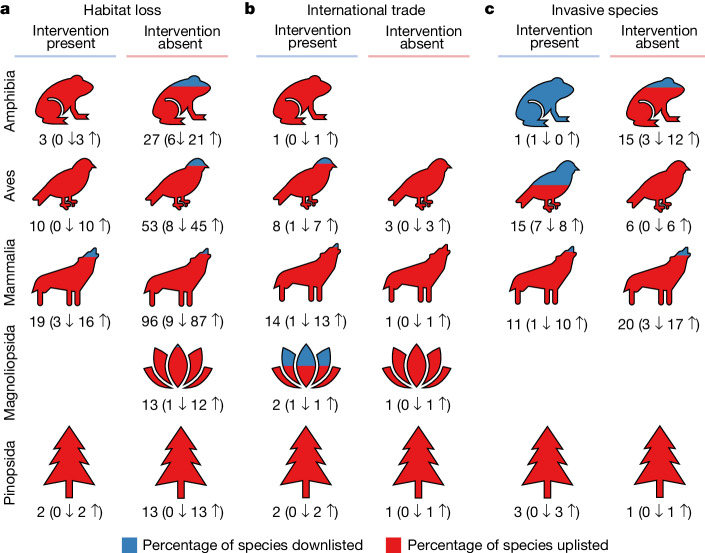
The percentage of species downlisted or uplisted to another threat category. **a**–**c**, We exclude ‘non-genuine’ status changes, which result from revisions in taxonomy or corrections of erroneous assessments. Columns show results for species with any one of habitat loss (**a**), overexploitation for international trade (**b**) or invasive species (**c**) listed as a major threat, where the appropriate type of conservation intervention is either documented as in place (left) or not (right). Numbers outside brackets represent the number of species changing Red List status, comprising the number downlisted (down arrow) plus the number uplisted (up arrow).

Although many threatened species are beneficiaries of documented conservation attention, whether those interventions work remains a critical question. From 2006 to 2020, 279 species were uplisted to a higher threat category and 41 species were downlisted to a lower threat category owing to a genuine increase and decrease in extinction risk, respectively (Extended Data Fig. 8). Of the downlisted species, only 15% (6 out of 41) lacked any documented conservation interventions. Alarmingly, 67% (187 out of 279) of uplisted species had received at least some documented conservation attention, suggesting that the measures used were insufficient to reverse declines. Focusing on species facing only one major threat (habitat loss, international trade or invasive species), a higher proportion were uplisted when the appropriate intervention was apparently lacking, although this was not consistently the case (Fig. 4). Thus, although conservation interventions are qualitatively associated with improvements in species’ Red List status, corroborating that conservation can succeed^8,9^, the mere existence of ‘something rather than nothing’ is not sufficient to reverse declines. Previous studies have documented large variation in how well conservation interventions are implemented, such as variation in PA management^34^. Additionally, ecological time lags occur in response to both positive and negative change^35^, and there are time lags inherent to the Red List process itself. Species must have met the criteria for a lower threat category for at least five years before the status change is implemented^36^. Few taxa were reassessed in the period 2006–2020, and birds were reassessed more frequently (approximately every 4 years) than any other group.

**Extended Data Fig. 5:**
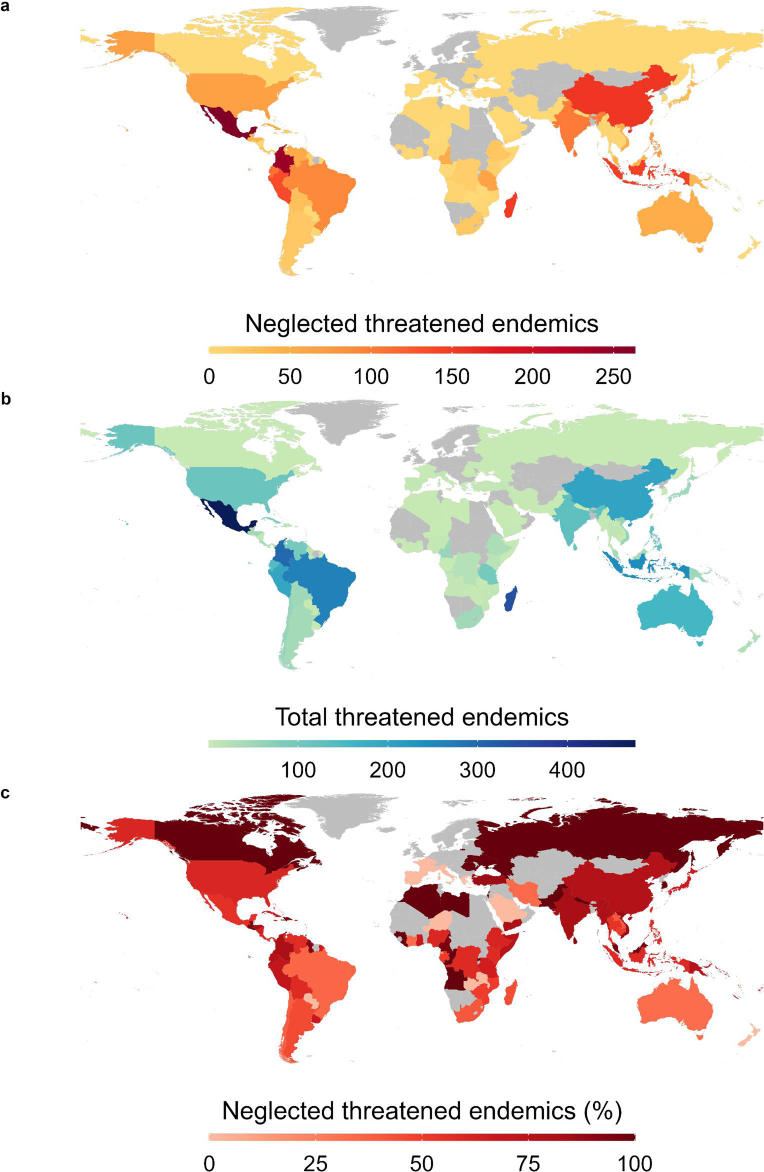
A breakdown of results for threatened endemic (i.e., found only in one country) species. Results are: (a) the total number of threatened endemic species lacking any documented conservation interventions; (b) the total number of threatened endemic species; and (c) the proportion of threatened endemic species within a country that are lacking any documented conservation interventions.

**Extended Data Fig. 6:**
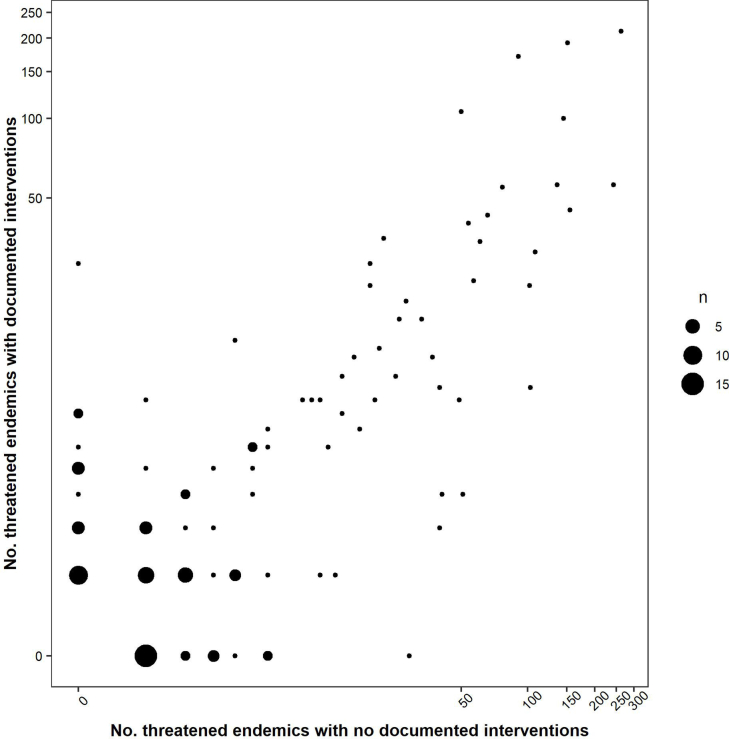
The number of threatened endemic (i.e., found only in one country) species per country with documented conservation interventions versus without documented interventions. Axes have been transformed to a log1p scale to aid visualisation. Point size denotes the number of observations at each location along the x and y axes.

**Extended Data Fig. 7:**
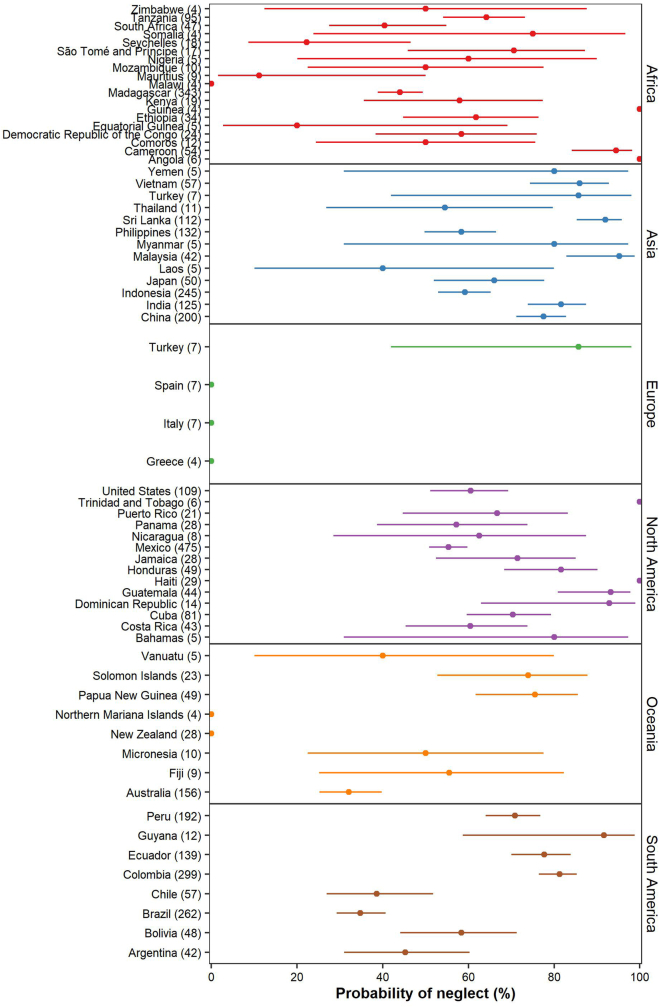
The model-predicted probability that endemic species in each country have no documented conservation interventions. Countries are shaded by continent (panels) to ease visual interpretation. Points are model-predicted fitted values with 95% confidence intervals. The sample size (n) is indicated in brackets after each country label.

**Extended Data Fig. 8:**
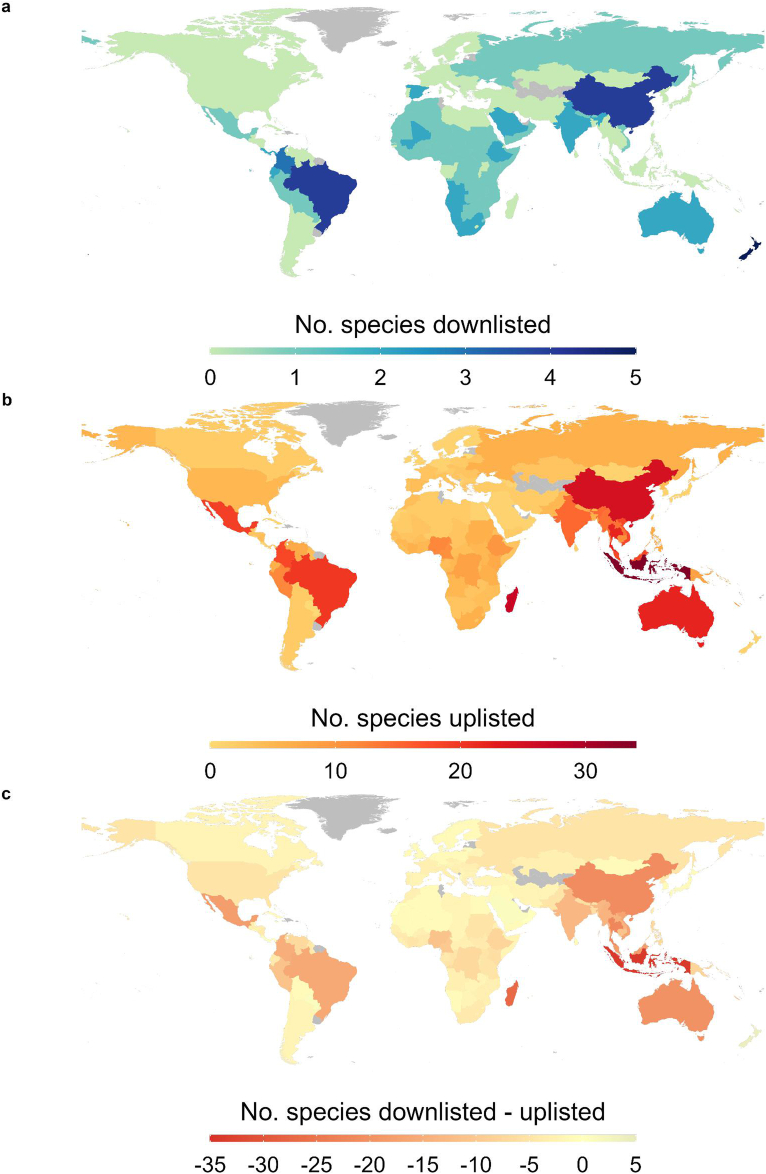
A breakdown of species that changed Red List status from 2006–2020 by country. Panels depict: (a) the total number of threatened species in a country that were downlisted; (b) the total number of threatened species in a country that were uplisted; and (c) the difference between the number of threatened species that were downlisted and the number that were uplisted. In panel (c), negative values (yellow to red) indicate that more species were uplisted than were downlisted, while the reverse is true for positive values (green).

**Extended Data Fig. 9:**
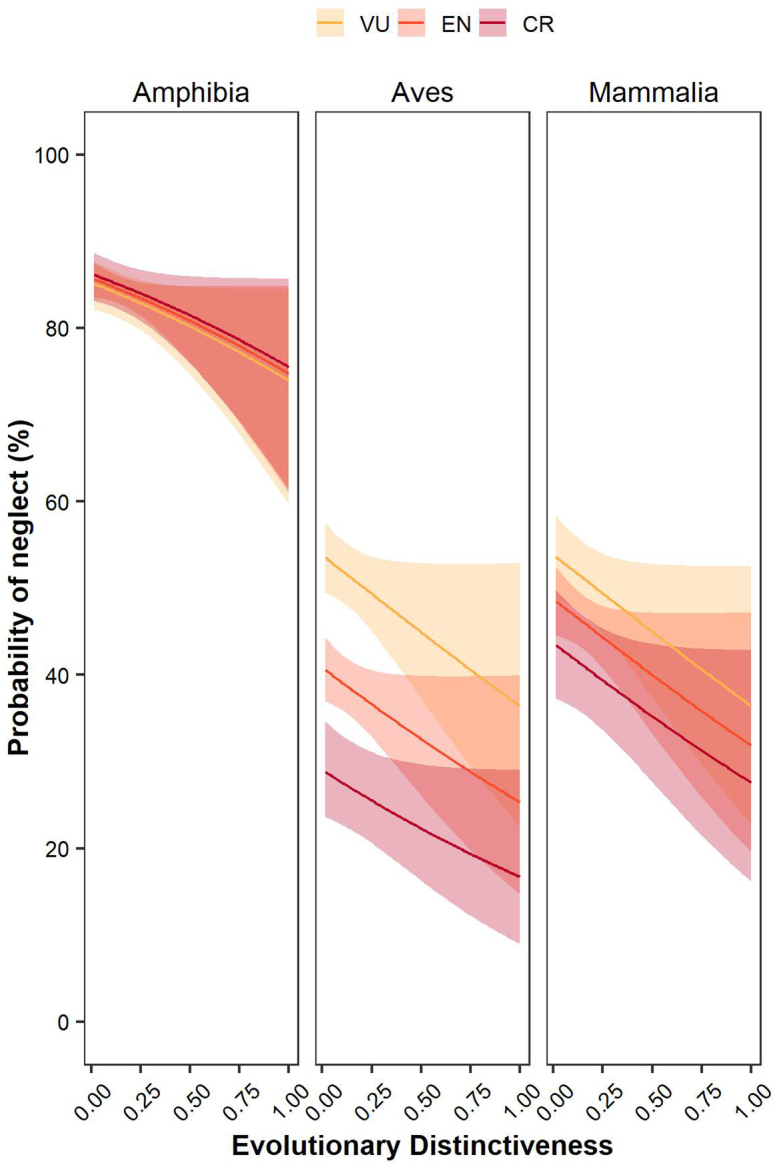
The model-predicted probability that threatened species have no documented conservation interventions, with increasing Evolutionary Distinctiveness. Panels separate taxonomic classes, and lines are coloured by Red List category: Vulnerable (yellow), Endangered (orange), and Critically Endangered (red). Shaded bands correspond to 95% Confidence Intervals. Evolutionary Distinctiveness is standardised within taxonomic classes to range between 0 and 1.

Given the geographic patterns in conservation attention, it is possible that certain groups of species are disproportionately neglected. We found a weak trend whereby ‘evolutionary distinctiveness’—a measure of species’ relative contribution to phylogenetic diversity^37^—was lower in species lacking meaningful habitat protection or any other documented conservation interventions, compared with species with at least one documented intervention (Extended Data Fig. 9; deviance = 4, d.f. = 1, *P* = 0.05). The total number of endemic threatened species with documented conservation interventions was positively correlated with the number that lacked such interventions (Extended Data Fig. 6). This may suggest that apparent neglect does not result from lack of will, but rather from insufficient capacity to act or to report for countries with greater numbers of threatened endemic species. However, the relationship was not straightforward (Extended Data Fig. 5), as the probability of apparent neglect for any given threatened endemic species was not statistically associated with the gross domestic product (GDP) of the country (deviance = 0, d.f. = 1, *P* > 0.05) or total number of endemic threatened species (deviance = 0, d.f. = 1, *P* > 0.05; Extended Data Fig. 7).

Teasing apart observed geographic patterns (Fig. 3) to discern causality requires a more nuanced assessment of conservation resources that additionally accounts for international aid and the sufficiency of interventions^34^. It is also important to note that even with detailed assessment guidelines and extensive training, geographic and taxonomic biases in assessor reporting exist^38^. This does not undermine our results, because it is crucial that scientists can identify where improved documentation is needed. It does, however, complicate interpretation, because we are not currently able to distinguish between a need for documentation and a true absence of conservation interventions.

Going forward, the IUCN Green Status of Species, which aims to track species recovery^39^, coupled with the expanding Conservation Evidence database^3^, should enable researchers to better explore the positive trends of some species on the Red List. Moreover, the commitment by more than 50 countries to expand the global PA network to encompass at least 30% of terrestrial and marine ecosystems by 2030 provides an opportunity to protect those species threatened by habitat loss that are currently under-represented in PAs^40^. Important sites for targeting expansion of PAs include Key Biodiversity Areas, most of which have been identified on the basis of the populations of threatened species they support^41^. Approaches that efficiently ensure adequate species representation will also be key to complement existing approaches for area-based conservation^42^. Outside of traditional PAs, other effective area-based conservation measures will have an important role in conserving threatened species^27,43^, especially on lands owned and managed by indigenous communities. However, a critical first step in improving global conservation practices is documenting what we are already doing. Globally, there are taxonomic and geographic biases in the species documented as receiving conservation attention, with limited instances of species being downlisted to lower categories of threat. Conservation can succeed, but without more and better targeted investment, we risk surrendering the world’s threatened species to mass extinction.

## Methods

### Data Sources

Information on species’ current status, threats and conservation interventions was downloaded from the Red List on 14 July 2020^1^. We focused only on terrestrial or terrestrial and freshwater species (as defined on the Red List) that are classified as threatened (Vulnerable, Endangered or Critically Endangered) and fall within the taxonomic groups that have been comprehensively assessed to at least family level (Supplementary Table 1): amphibians (2,220), birds (1,295), cycads (192), mammals (1,258), conifers (198), crocodiles and alligators (9), birches (19), magnolias (148), southern beeches (11), teas (88), cacti (415), freshwater crabs (36) and chameleons (74). This gave a total of 5,963 species. We reclassified threats into six broader categories (Supplementary Table 2), in line with those listed by the Convention on Biological Diversity^44^: (1) habitat loss (including habitat degradation); (2) overexploitation; (3) pollution; (4) invasive alien species; (5) climate change; and (6) other (for example, unknown threats and threats due to natural causes, such as volcanic eruptions).

For the broad category of habitat loss, we further required that the threat be coded, according to the Red List ‘stresses’ classification scheme, as an ‘ecosystem/community stress’. This includes ecosystem stresses in the form of ecosystem conversion, ecosystem degradation and indirect ecosystem effects (such as ecosystem fragmentation), but does not include stresses that solely and directly affect the species. We applied this additional restriction because some of the threats that could potentially drive habitat loss, such as ‘recreational activities’ (Supplementary Table 2), could instead cause species stresses such as direct mortality, competition and reduced reproductive success, while leaving habitats intact.

We restricted our analysis of the Convention on Biological Diversity threat ‘overexploitation’ to cases involving the intentional use of internationally traded species because this is a major known threat to biodiversity that can be clearly matched to the conservation intervention ‘international trade control’^45,46^. Other forms of resource use, such as ‘local, subsistence hunting’, fall into the threat category ‘other’.

In all analyses, we focus only on threats that we deemed to have a major influence on a species’ risk of extinction, based on the IUCN-defined fields: ‘scope’, ‘severity’ and ‘timing’. Specifically, the threat must: (1) affect more than 50% of the global population of a species; (2) have caused ‘slow, significant declines’, ‘rapid declines’ or ‘very rapid declines’ in the population; and (3) have occurred in the past or be ongoing (that is, not a predicted future threat). We also include cases where the scope or severity of the threat is unknown because, depending on the threat category, between 488 and 4,410 threatened species had one of the six threats listed but with unknown scope and/or severity. Excluding cases where threat severity or scope was unknown did not affect our conclusions (Supplementary Figs. 6 and 7).

Of the 1,300 species in our database that are categorized on the Red List as Critically Endangered, 179 are Possibly Extinct. Species that are Possibly Extinct are potentially less likely to have conservation interventions in place, since conservationists may not know where the species occurs or what interventions it needs. Moreover, such species may be subject to triage due to the low chance of conservation success. However, we found that our results were robust to excluding Possibly Extinct species (Supplementary Figs.  6 and 7).

For each species, the Red List denotes various conservation interventions as either in place or not. Our focus was on the following six interventions, as they are defined in the Red List ‘conservation actions in place’ classification scheme: (1) Does the taxon occur in at least one PA?; (2) Is the taxon subject to any international management/trade controls?; (3) Is there invasive species control or prevention?; (4) Has the taxon been successfully reintroduced or introduced benignly?; (5) Is the taxon included in international legislation?; and (6) Is the taxon the subject of any recent education or awareness programmes?.

We did not consider the intervention ‘ex situ conservation’, in place for 681 species in our dataset, because we are interested in actions focused on decreasing species’ risk of extinction in the wild (although we recognize that ex situ conservation can ultimately contribute to the recovery of threatened species in the wild).

Although we have adopted the Red List classification scheme here, and we use Red List data to detect the presence of most conservation interventions, in our main analyses we chose not to use Red List data to determine whether a species occurs in PAs. The Red List’s binary classification of whether a species is present in PAs is determined by published and unpublished literature, in combination with expert knowledge of the distribution of populations, rather than by using a representation threshold. This allows a species to be categorized as receiving habitat protection if just a small fraction of its habitat falls within the boundaries of a single PA. Instead, we use the Species Protection Score, which is used in the calculation of Species Protection Index—one of the indicator metrics for species representation within the Kunming–Montreal Global Biodiversity Framework^21,25^ (https://mol.org/indicators; Supplementary Fig. 1). Species Protection Scores are calculated as the percentage of each species’ range that occurs within the boundaries of PAs (WDPA)^22^, relative to a pre-determined, species-specific representation threshold. For example, if 50% of a species range occurs within PAs and the representation threshold for that species is 50%, then the Species Protection Score is 100. Conversely, if the representation threshold for that species is 80%, then a 50% overlap of the species’ range with PAs would correspond to a Species Protection Score of only 62.5 (that is, (50/80) × 100). While work on a more ecologically fine-tuned yet broadly applicable determination of representation thresholds is in progress, the threshold itself is adapted from Rodrigues et al.^47^, whereby we specify that species with less than or equal to 10,000 km^2^ habitat must have 100% of that habitat occurring within PAs for the species to be considered meaningfully represented in PAs. Species with more than or equal to 250,000 km^2^ habitat must have at least 15% of that habitat occurring within PAs for the species to be considered meaningfully protected by PAs. For all other values of range size (10,000 km^2^ ≤ range ≤ 250,000 km^2^), a log-linear interpolation between 15 and 100 is applied to calculate the appropriate representation threshold. These thresholds assume that species with less habitat require a greater proportion of that habitat to occur within PAs for those species to be considered protected from habitat loss and degradation.

In this study, we assigned a binary value to the Species Protection Score such that the species was considered to be meaningfully represented in PAs only if its Species Protection Score was 100. All other values of the Species Protection Score indicate a failure to achieve meaningful representation of that species within the existing PA network. We note that the calculation of Species Protection Scores can be performed in two ways, depending on the range maps available. All species on Map of Life have expert map ranges, and thus all species had Species Protection Scores derived from overlapping these expert map ranges with PAs. In addition, most species also had Species Protection Scores based on overlapping PAs with ‘habitat-suitable ranges’ (HSRs), whereby each species’ range is refined according to its habitat and elevation preferences^48^ (also known as ‘area of habitat’). To be conservative, we considered a species to be meaningfully represented in PAs if either of these Species Protection Scores was 100. To explore species’ HSR results and Protection Scores, see http://mol.org/indicators/protection.

For independent validation of the Species Protection Score, we also applied our own analogous protocol to derive HSR for each species and calculate its percentage overlap with the WDPA. Species-specific representation thresholds were calculated as above. HSR could be calculated for most of the species in our dataset, because we focus on the taxonomic families that have spatial range data available from the Red List^1^ and BirdLife International (http://datazone.birdlife.org/home), including all bird, mammal and amphibian families, plus select families of reptiles (Chamaeleonidae, Crocodylidae and Gavialidae), and flowering plants (Theaceae, Magnoliaceae, Betulaceae and Nothofagaceae). The WDPA was cleaned following best-practice guidelines^49,50^. All spatial analyses were conducted in Google Earth Engine^51^. Following, Powers and Jetz^48^ and Brooks et al.^52^, HSR was determined by refining range polygons to areas with suitable land cover and elevation. Habitat preferences and elevation limits for each species were obtained from the Red List^1^ and Quintero and Jetz^53^. For elevation we use the EarthEnv Digital Elevation Model version 1^54^, resampled from ~90 m to 1 km. For land cover we use a map of terrestrial habitat types for the year 2015, specifically designed to match the habitat classification scheme of the IUCN^55^. Where a species’ habitat preference is given only to the coarser level 1 classification (for example, ‘Forest’), we conservatively assume that all nested level 2 categories are also suitable. We use the fractional habitat types map aggregated to 1 km resolution, following the recommendation of Jung et al.^55^.

In our calculation of HSR, limitations of the input data resulted in 621 species with zero HSR. In these cases, we additionally verified if that species occurs in PAs if it had been observed within a PA in the last five years (Supplementary Fig. 1), based on point occurrence records from the Global Biodiversity Information Facility (GBIF)^56^. GBIF records were retrieved in R version 4.3.2^57^, using the packages rgbif^58^ and taxize^59^, and points were buffered by 300 m to allow for positional errors^55^. By repeating the analyses of the main text with our independent calculation of HSR, we find very similar results to the main analyses based on the Species Protection Score (Supplementary Figs. 2–5). However, if we use a less conservative approach whereby the determination of whether a species occurs in PAs is based on both the Red List data and threshold percentage overlap of HSR with PAs (Supplementary Figs. 2–5), a far greater number of species appear to have meaningful habitat protection, and therefore a greater number also appear to have at least one documented conservation intervention.

Preliminary checks suggested that conservation intervention information is lacking for many assessed species in the Red List data download, despite being evident in the detailed text description of species’ Red List profiles. As a result, we supplemented the binary classification provided in the tabular data download by manually reviewing the ‘Conservation Actions In Place’ text for each threatened species (n = 5,963), using the same criteria as defined by IUCN^60^. Thus, the first test of whether a conservation intervention is in place is whether it is recorded as such in the Red List tabular data. If not, the second test is whether the intervention is described as in place in the Red List text description. Failing both tests, the conservation intervention is recorded as not in place, except for the subset of interventions with additional or alternative sources of information outside of the Red List. These interventions were: PA coverage (as described above), international trade control and legislation, and invasive species control. The details of the additional tests are described in detail below and in Supplementary Fig. 1.

International legislation includes international trade control, hence only one was used in any given analysis (that is, either international legislation or international trade control). Most of our analyses focus on interventions matched to one of three major threats, hence we use international trade control as the appropriate intervention for the threat of overexploitation for international trade. We consider a species to be subject to international trade control only if stated as such on the Red List, or if the species (or a larger taxonomic group of which it is part) is listed on any of the appendices of the Convention on International Trade in Endangered Species of Wild Fauna and Flora (CITES) (Supplementary Fig. 1).

Several additional conservation interventions exist that are less targeted to a specific threat, but which are, nevertheless, important tools for reducing species’ overall risk of extinction^17^. Thus, we include an additional analysis of how many species have any documented conservation interventions, irrespective of what their main threats are. The scope of this analysis is broader, hence we replace the intervention of international trade control with the broader intervention of international legislation, which additionally includes multilateral agreements that are not directly concerned with trade control, such as the Convention on the Conservation of Migratory Species of Wild Animals (CMS). Specifically, we consider species to be subject to international legislation if they meet any one of the following criteria (Supplementary Fig. 1): (1) The species is subject to international trade control as defined above (stated as such on the Red List, or the species is listed on any of the CITES appendices); (2) The species is listed on published legislation listings from Species+, which includes all species that are listed in the Appendices of CITES and CMS, as well as species listed in the Annexes to the EU Wildlife Trade Regulations; or (3) The species is described as subject to a named multilateral agreement (see Supplementary Table 4 for legislation considered).

In addition to the described calculation of HSR overlap with PAs, we also overlaid HSR with spatial data available for invasive species eradication efforts (DIISE)^23^. For island species at risk from invasive vertebrates, we identified cases where any of the threatened species’ HSR overlapped with an island from which its threatening invasive species had been successfully eradicated^23^ (Supplementary Fig. 1). This determination was based only on eradication of vertebrate species specifically named by the Red List as affecting the threatened species in question. Threatened island species were identified as those threatened species with more than 95% of their range area occurring on islands, based on the overlap between island polygons from the Global Island Database^61^, and species’ historical range (the sum of polygons with presence codes: ‘extant’, ‘probably extant’, ‘possibly extinct’, extinct’ and ‘presence uncertain’) (http://datazone.birdlife.org/home). We focus on threatened island species only, because spatial data for invasive species eradication efforts are reliably available only for islands^23^. We note that wide-ranging threatened species may also have part of their range on islands, where they, too, may be threatened by invasive island vertebrates. However, with our data sources it is not possible to determine precisely where in a species’ range a named invasive vertebrate is exerting its impact, and thus we can only be sure that a threatened species benefits from eradication efforts on islands if that threatened species is itself an island endemic.

To determine if there are geographic hotspots of apparent conservation neglect, we mapped the spatial distribution of threatened species that lack documented conservation interventions. We determined the countries in which each species occurs using information from Map of Life. The steps described above identified the species lacking appropriate types of conservation intervention to tackle the three major threats that we assessed, as well as the species lacking any documented conservation interventions. In both cases (separately), we created maps by summing the number of apparently neglected species occurring in each country. In statistical analyses we focus on country endemics to allow us to more precisely pinpoint where conservation effort is apparently lacking. We include all species in the maps (except for Extended Data Fig. 5), but note that the conservation interventions data provided by the Red List are not spatially explicit. As such, the presence or absence of conservation interventions is assigned to all parts of a species’ global distribution. For non-endemics, there are instances where a species appears to lack a particular intervention in all countries where it occurs, but in fact the intervention is only necessary in a subset of countries. Conversely, there are instances where a species appears to benefit from an intervention throughout its entire range, but in reality the intervention is implemented in only one of the countries in which it occurs.

To qualitatively explore whether documented conservation interventions were associated with species’ risk of extinction, we focused on species that have changed Red List status. Any official change in Red List status is the result of extensive assessment by IUCN. Status change tables covering the years 2006–2020 were downloaded on 14 July 2020^62^. We excluded records where the change in status of a given species was due to non-biological factors, such as new information or a change in taxonomy. We also excluded cases where an allegedly ‘genuine’ status change was later superseded by a non-genuine change of status in the opposing direction. For example, in 2008 the Mauritian flying fox (*Pteropus niger*) was listed as having genuinely improved in status, moving from Endangered to Vulnerable, but in 2013 the species was uplisted back to Endangered because previous assessments were found to have used incorrect data. For species genuinely changing status multiple times, we use only the first instance.

### Analyses

All analyses were performed in R version 4.3.2^57^. Full model results are reported in Supplementary Tables 2–5. Model inference was made using likelihood ratio tests, dropping each variable in turn and comparing the reduced model to the full model^63^. We used generalized linear models (GLMs) with a binomial error structure, fit using the glm function of the lme4 package^64^, to model both the proportion of species documented as receiving the appropriate type of conservation intervention, and the proportion of species with no documented interventions. In the former, each threat with a matched conservation intervention was modelled separately, to avoid double-counting species facing multiple threats. Explanatory variables were taxonomic class and Red List category, and the interaction between them.

We additionally tested whether the number of species documented as receiving conservation attention differed between countries. The two response variables tested were: the proportion of species in each country documented as receiving the appropriate type of conservation intervention (tested for each threat separately); and the proportion of species in each country with no documented interventions. Both response variables were modelled against the explanatory variable of country, again using GLMs with a binomial error structure. Subsequently, to explore the drivers of country-level conservation effort, we tested whether the modelled probability of a species receiving no documented conservation interventions was predicted by country GDP^65^ or the total number of threatened endemic species occurring in that country.

We might expect that instances of species changing Red List status would be associated with both: (1) the presence or absence of the type of conservation intervention appropriate to tackle the three major threats that we assessed; and (2) the presence/absence of any documented conservation interventions. In the first case, we considered the subset of species changing Red List status that had either habitat loss, overexploitation for international trade, or invasive species, as their only major threat of the three threats that we assessed. We summarized the number of species uplisted and downlisted according to whether they were documented as receiving the appropriate type of conservation intervention to tackle the one major threat they faced (out of the three threats that we assessed). We consider the appropriate type of intervention for these three major threats to be (respectively): occurring within a PA; international trade control; and invasive species control. For the second question, we summarized the number of species being uplisted to a higher threat category, or downlisted to a lower threat category, according to whether any conservation interventions had been documented for those species.

Finally, we explored the potential consequences on phylogenetic diversity of apparent biases in conservation effort, by modelling the proportion of all amphibian, bird and mammal species with and without documented conservation interventions, according to their evolutionary distinctiveness^37^. Evolutionary distinctiveness is a measure of species’ relative contribution to phylogenetic diversity^37^. Evolutionary distinctiveness data were downloaded from EDGE of Existence (https://www.edgeofexistence.org/edge-lists/). We used a GLM with a binomial error structure, including both Red List category and evolutionary distinctiveness as explanatory variables. The latter was standardized within each taxonomic class to range between 0 and 1.

### R Packages

Data wrangling: dplyr^66^, pdftools^67^, purrr^68^, reticulate^69^, tidyr^70^. GBIF data download: rgbif^58^, taxize^59^. Spatial data: sf^71^. Statistical analyses: lme4^64^. Data visualization: cowplot^72^, DiagrammeR^73^, DiagrammeRsvg^74^, egg^75^, ggplot2^76^, ggnewscale^77^, ggtext^78^, gridExtra^79^, png^80^, RColorBrewer^81^, rphylopic^82^, rsvg^83^, scales^84^. Document preparation: bookdown^85^, kableExtra^86^, knitr^87^.

### Reporting summary

Further information on research design is available in the Nature Portfolio Reporting Summary linked to this article.

## Online content

Any methods, additional references, Nature Portfolio reporting summaries, source data, extended data, supplementary information, acknowledgements, peer review information; details of author contributions and competing interests; and statements of data and code availability are available at 10.1038/s41586-024-07498-7.

### Supplementary information

Supplementary Information This file contains Supplementary Figs. 1–7, Supplementary Tables 1–5 and supplementary references.

Reporting Summary

Supplementary Data 1 Data for all 5,963 threatened species including in our analyses of conservation interventions.

Supplementary Data 2 Data for all 320 threatened species including in our analyses of changes in Red List category.

Supplementary Data 3 Metadata for Supplementary Data 1 and 2.

Supplementary Data 4 Reclassification of IUCN threat categories into simplified categories based on the five main threats recognized by the Convention on Biological Diversity.

Supplementary Data 5 Conservation legislation considered when determining if a species is subject to international legislation. Compiled from multilateral agreements named in the ‘Conservation Actions In Place’ text downloaded from the IUCN Red List.

Supplementary Data 6 Summary of species lacking appropriate conservation intervention, in each country and for each of the three major threats to biodiversity with a clearly matched intervention: habitat loss (a), overexploitation for international trade (b) and invasive species (c).

Supplementary Data 7 The number of species changing Red List status from 2006–2020.

Supplementary Data 8 Credit and source details for the PhyloPic silhouettes used in the figures.

## Data Availability

Processed data to support the findings presented here are available as Supplementary Data  1–3 and on Zenodo (10.5281/zenodo.10813823)^88^. The original source datasets are available for download by request from their respective providers. Species data: species assessments can be requested from the IUCN Red List of Threatened Species website at https://www.iucnredlist.org; status change tables are available in pdf format in table 7 at https://nc.iucnredlist.org/redlist/content/attachment_files/Table_7_2007-2022.zip; range maps can be requested at https://www.iucnredlist.org/resources/spatial-data-download; Species Protection Scores can be requested from Map of Life at https://mol.org/species/; elevation preferences for birds^53^ are available at https://static-content.springer.com/esm/art%3A10.1038%2Fnature25794/MediaObjects/41586_2018_BFnature25794_MOESM3_ESM.xlsx; point occurrence records for species with zero HSR were downloaded from the GBIF^56^ at 10.15468/DL.DVP728; EDGE data are available at https://www.edgeofexistence.org/wp-content/uploads/2023/12/2023_EDGE_species_RT_call.xlsx. Conservation interventions data: PA boundaries can be requested from the WDPA at https://www.protectedplanet.net/en/thematic-areas/wdpa?tab=WDPA; The DIISE^23^ is available at http://diise.islandconservation.org/; international trade control data (CITES, CMS and EU Annexes) are available from Species+ at https://speciesplus.net/. Geographic data: Global Administrative Areas are available at https://gadm.org/data.html; the Global Islands Database^89^ is available at https://resources.unep-wcmc.org/products/f98e179ec3f448e59dfe9bda248ff4b6; elevation was derived from the EarthEnv Digital Elevation Model version 1 (ref. ^56^), available at https://www.earthenv.org/DEM; the global terrestrial habitat types map^55^ is available at https://zenodo.org/records/4058819; country GDP is available at https://data.worldbank.org/indicator/NY.GDP.MKTP.CD?locations=1W.

## Code availability

R code to reproduce the results and manuscript is available on GitHub at https://github.com/rasenior/ConservationActions, and is also packaged with the data on Zenodo (10.5281/zenodo.10813823)^88^.
