# Supplementary material for: Global shortfalls in documented actions to conserve biodiversity
Source: Nature. 2024 Jun 5;630(8016):387–91. doi: 10.1038/s41586-024-07498-7 (PMC11168922; doi:10.1038/s41586-024-07498-7)
Supplement: Supplementary file 1 — This file contains Supplementary Figs. 1–7, Supplementary Tables 1–5 and supplementary references. [file 41586_2024_7498_MOESM1_ESM.pdf]

nature portfolio

https://doi.org/10.1038/s41586-024-07498-7

Supplementary information

Global shortfalls in documented actions to conserve biodiversity

In the format provided by the
authors and unedited

Global shortfalls in documented actions to conserve biodiversity

Supplementary Information

Rebecca A. Senior[1,2]*, Ruby Bagwyn[3], Danyan Leng[4,5], Alexander K. Killion[4,5], Walter Jetz[4,5], and David S. Wilcove[1,6]

[1]Princeton School of Public and International Affairs, Princeton University, Princeton, NJ, USA.

[2]Conservation Ecology Group, Department of Biosciences, Durham University, Durham DH1 3LE, UK

[3]Williams College, Williamstown, MA, USA.

[4]Department of Ecology & Evolutionary Biology, Yale University, New Haven, CT, USA.

[5]Center for Biodiversity and Global Change, Yale University, New Haven, CT, USA.

[6]Department of Ecology and Evolutionary Biology, Princeton University, Princeton, NJ, USA.

***Corresponding author:** rebecca.a.senior@gmail.com (R.A. Senior)

Contents

1 Supplementary Figures **4**

2 Supplementary Tables **11**

Supplementary References **12**

1 Supplementary Figures

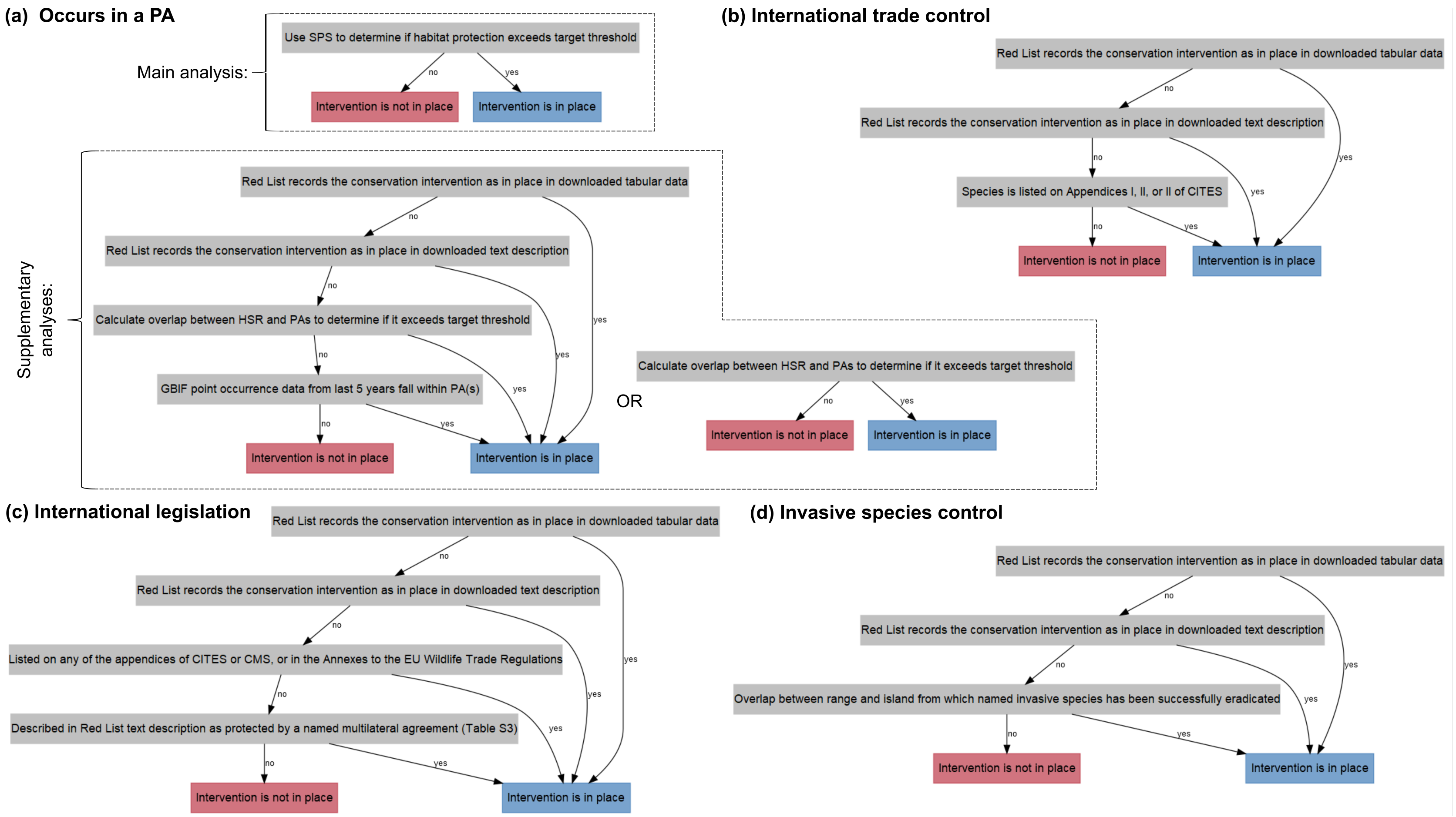

Supplementary Figure 1: Schematic diagram of the protocol for defining conservation interventions as in place or not.

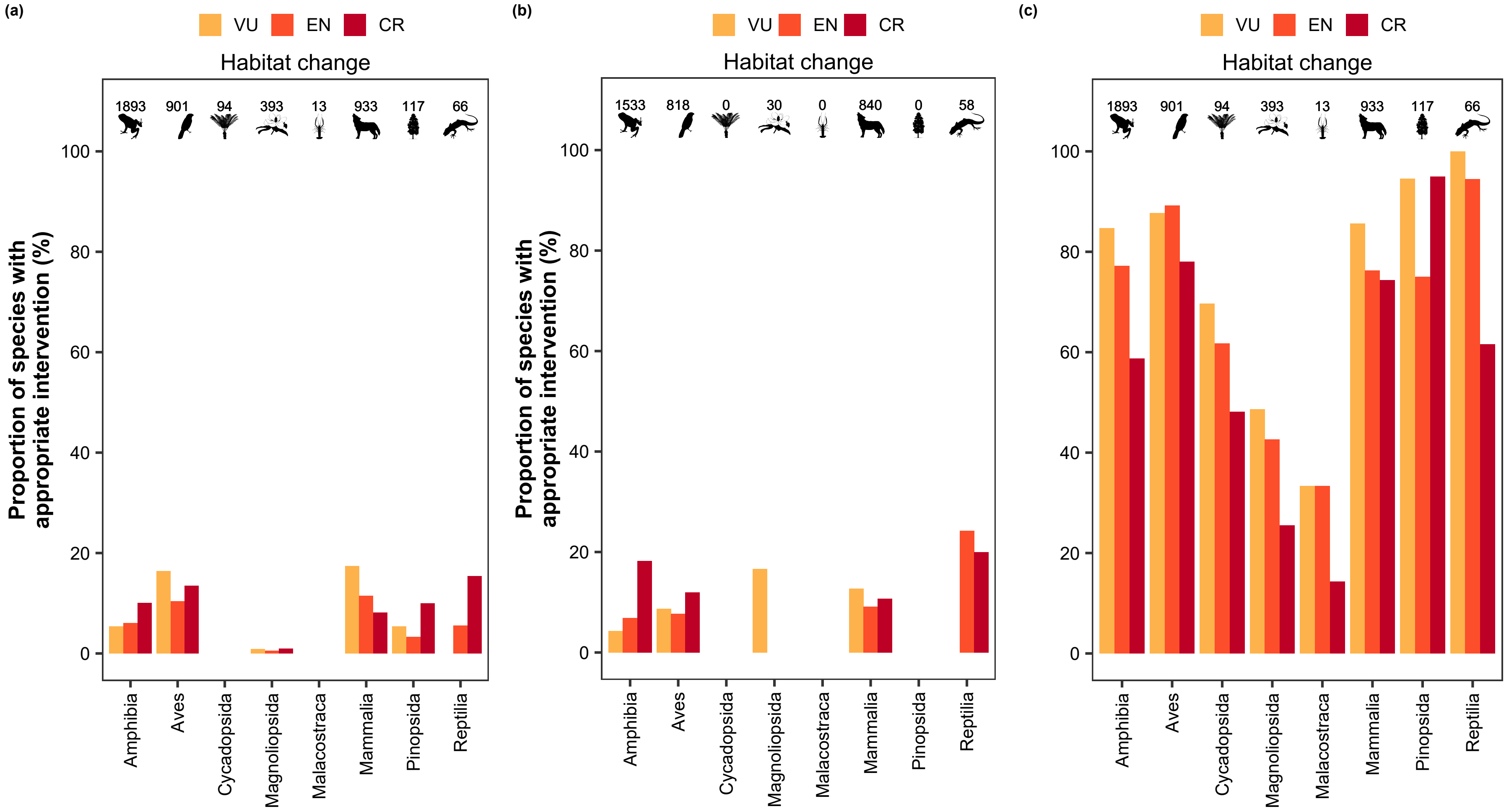

Supplementary Figure 2: The proportion of species documented as threatened by habitat loss and occurring in PAs. Panel (a) represents the results from the main analyses (Figure 1), where the Species Protection Score (Jetz et al., 2022) is used to determine whether species occur in PAs (Figure S1a). For comparison, panel (b) uses an independent but analogous process of overlapping species' Area of Habitat with PAs, and comparing this percentage overlap against species-specific representation thresholds to determine which species occur in PAs (see Methods). The same is done in panel (c), but after first giving precedence to whether the Red List identifies the species as occurring in PAs. Different bar colours denote different Red List categories: Vulnerable (VU; yellow), Endangered (EN; orange), and Critically Endangered (CR; red). Small numbers above the bars represent the total number of species included in our analyses, by taxonomic class.

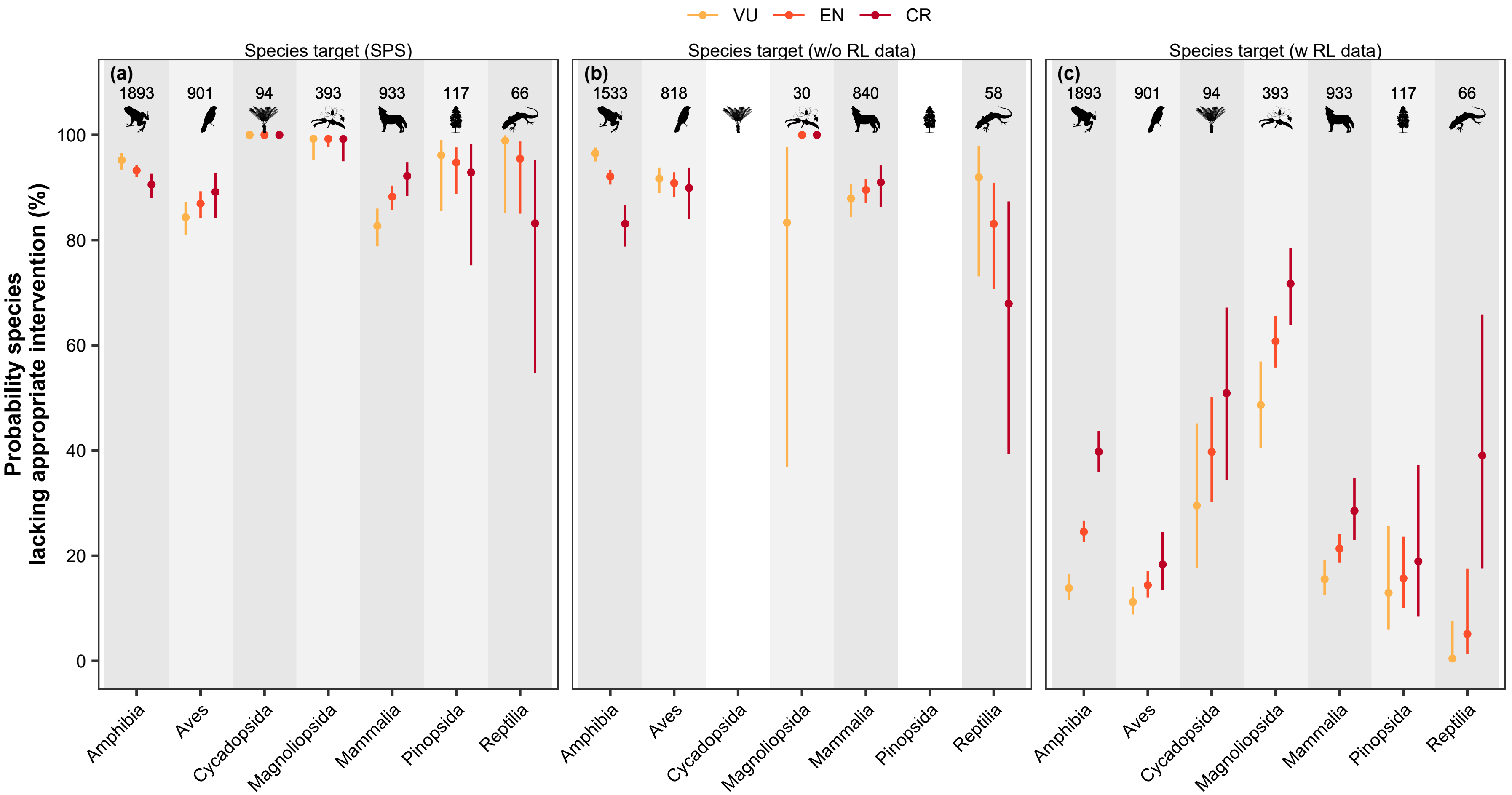

Supplementary Figure 3: The model-predicted probability of species being documented as threatened by habitat loss and occurring in PAs. Panel (a) represents the results from the main analyses (Figure 1), where the Species Protection Score (Jetz et al., 2022) is used to determine whether species occur in PAs (Figure S1a). For comparison, panel (b) uses an independent but analogous process of overlapping species' Area of Habitat with PAs, and comparing this percentage overlap against species-specific representation thresholds to determine which species occur in PAs (see Methods). The same is done in panel (c), but after first giving precedence to whether the Red List identifies the species as occurring in PAs. Point colours denote different Red List categories: Vulnerable (VU; yellow), Endangered (EN; orange), and Critically Endangered (CR; red). Small numbers at the top of the panels denote the sample size. Points are model-predicted fitted values with 95% confidence intervals.

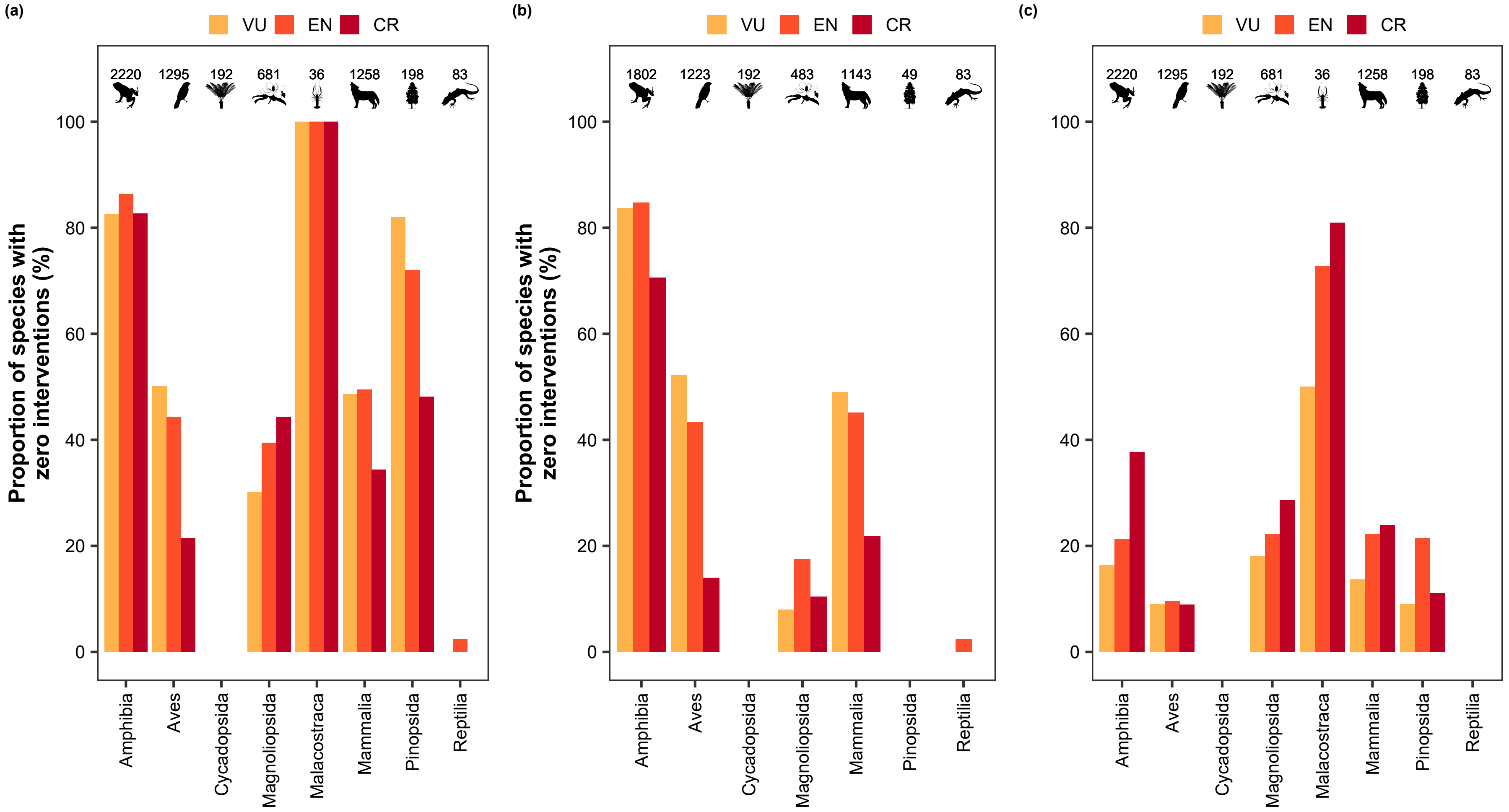

Supplementary Figure 4: The proportion of threatened species with no documentation of implementation of any of the six categories of conservation intervention (irrespective of threat). Panel (a) represents the results from the main analyses (Figure 1), where the Species Protection Score (Jetz et al., 2022) is used to determine whether species occur in PAs (Figure S1a). For comparison, panel (b) uses an independent but analogous process of overlapping species' Area of Habitat with PAs, and comparing this percentage overlap against species-specific representation thresholds to determine which species occur in PAs (see Methods). The same is done in panel (c), but after first giving precedence to whether the Red List identifies the species as occurring in PAs. Different bar colours denote different Red List categories: Vulnerable (yellow), Endangered (orange), and Critically Endangered (red). Small numbers above the bars represent the total number of species included in our analyses, by taxonomic class.

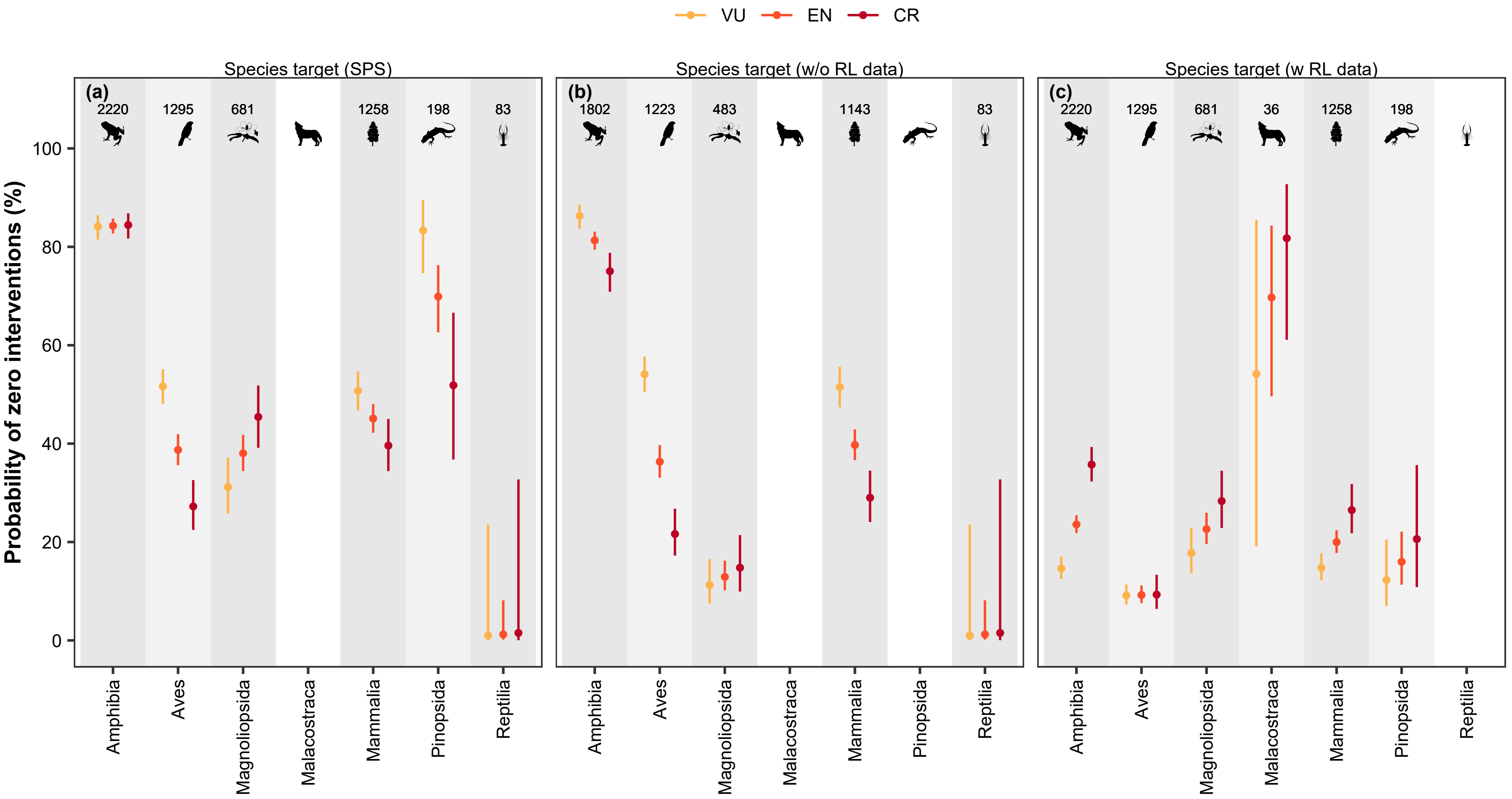

Supplementary Figure 5: The model-predicted probability of threatened species receiving any documented conservation intervention. Panel (a) represents the results from the main analyses (Figure 1), where the Species Protection Score(Jetz et al., 2022) is used to determine whether species occur in PAs (Figure S1a). For comparison, panel (b) uses an independent but analogous process of overlapping species' Area of Habitat with PAs, and comparing this percentage overlap against species-specific representation thresholds to determine which species occur in PAs (see Methods). The same is done in panel (c), but after first giving precedence to whether the Red List identifies the species as occurring in PAs. Point colours denote different Red List categories: Vulnerable (VU; yellow), Endangered (EN; orange), and Critically Endangered (CR; red). Small numbers at the top of the panels denote the sample size. Points are model-predicted fitted values with 95% confidence intervals.

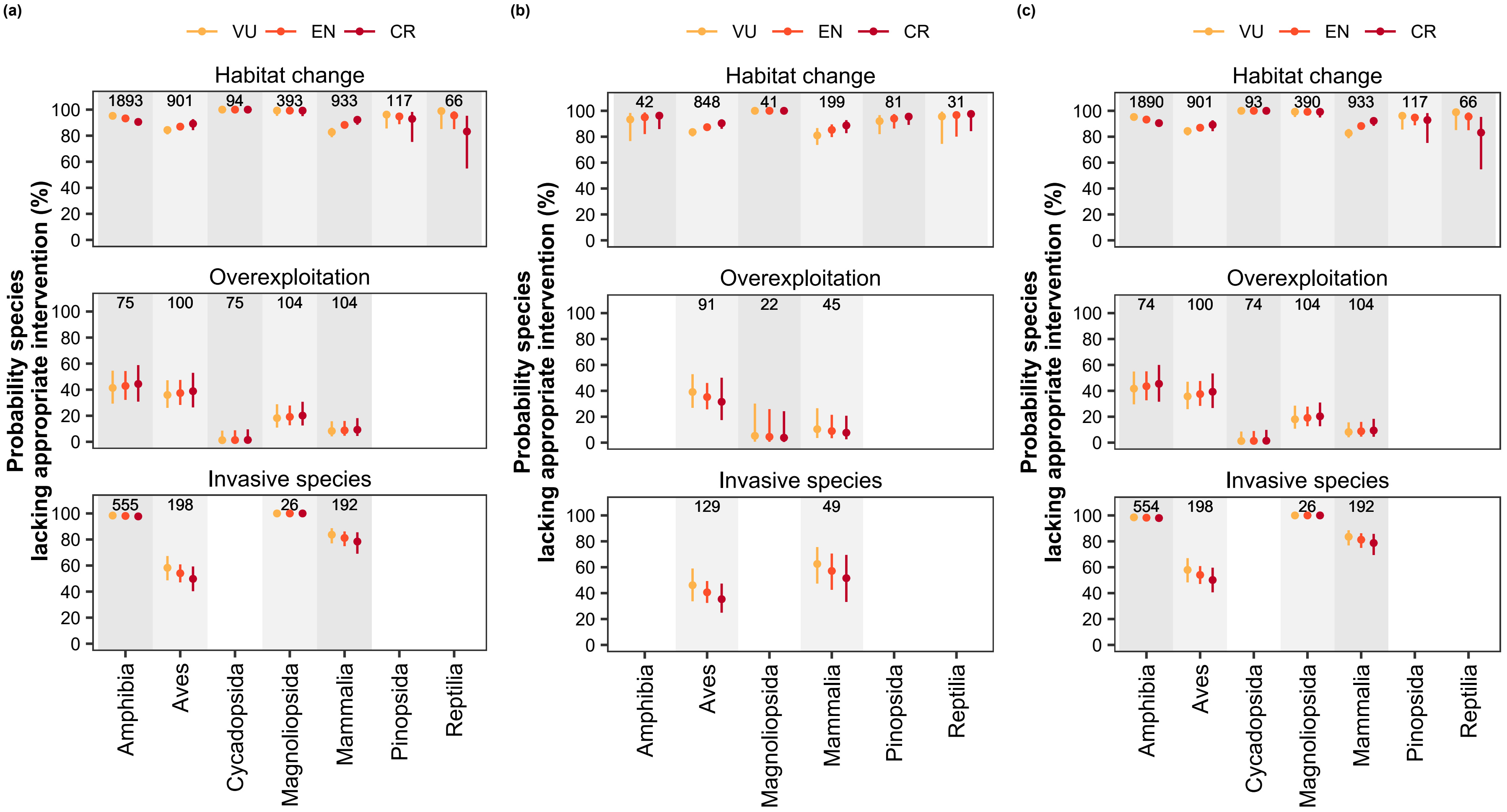

Supplementary Figure 6: The model-predicted probability of threatened species receiving the appropriate conservation intervention. Results are shown for the three major threats to biodiversity with a clearly matched intervention (rows). Column (a) depicts results from the main analyses. Column (b) depicts results when excluding cases where the scope or severity of the threat is unknown. Column (c) depicts results when excluding species that are listed as “Possibly Extinct”. Point colours denote different Red List categories: Vulnerable (yellow), Endangered (orange), and Critically Endangered (red). Small numbers at the top of the panels denote the sample size. Points are model-predicted fitted values with 95% confidence intervals.

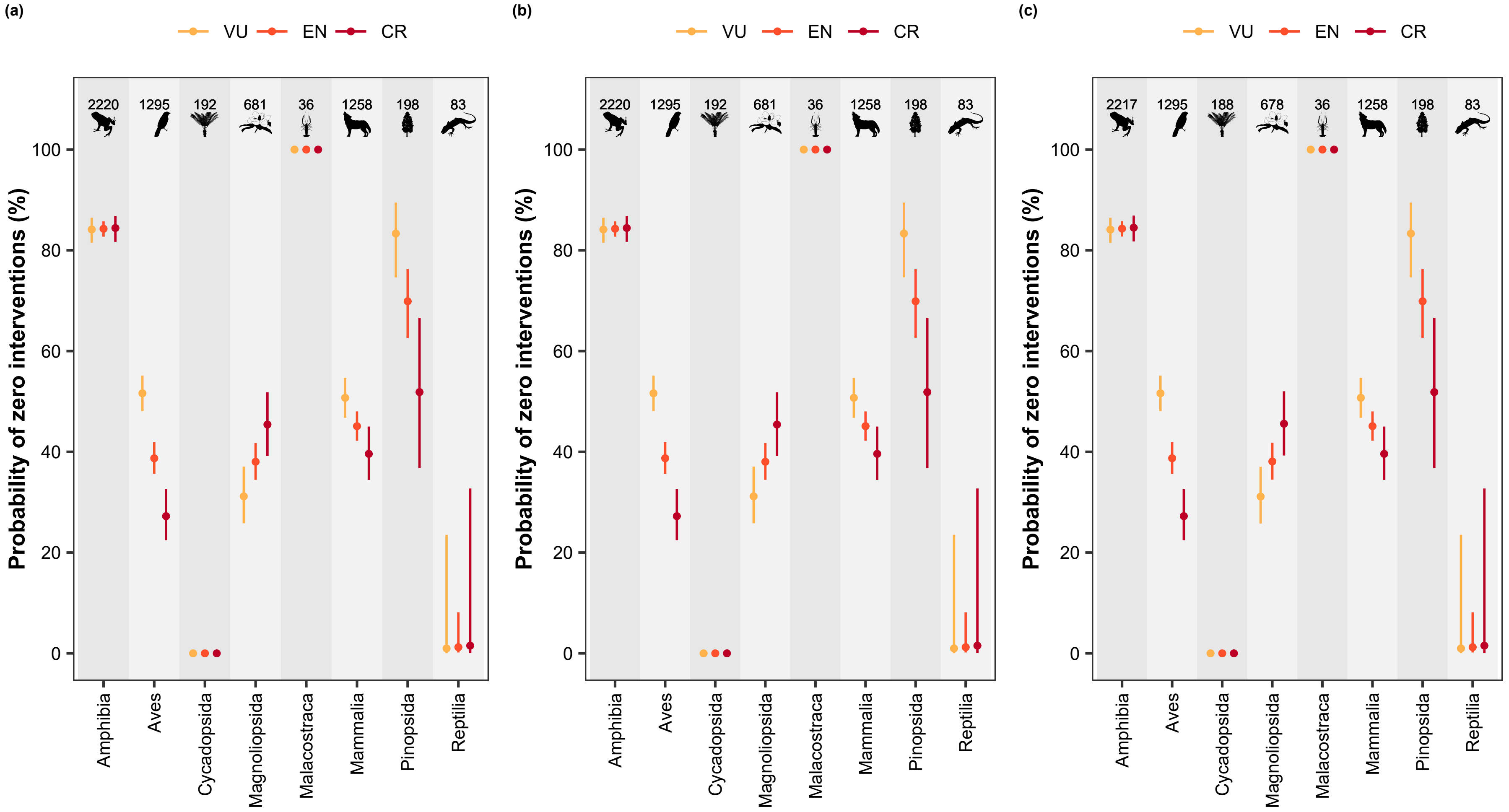

Supplementary Figure 7: The model-predicted probability of threatened species receiving any documented conservation intervention. Column (a) depicts results from the main analyses. Column (b) depicts results when excluding cases where the scope or severity of the threat is unknown. Column (c) depicts results when excluding species that are listed as “Possibly Extinct”. Point colours denote different Red List categories: Vulnerable (yellow), Endangered (orange), and Critically Endangered (red). Small numbers at the top of the panels denote the sample size. Points are model-predicted fitted values with 95% confidence intervals.

2 Supplementary Tables

Supplementary Table 1: Taxa comprehensively assessed on the IUCN Red List and included in our main analyses.

Common name	Scientific name	Taxonomic level	Taxonomic class	Total species
Amphibians	Amphibia	Class	Amphibia	2220
Birds	Aves	Class	Aves	1295
Cycads	Cycadopsida	Class	Cycadopsida	192
Mammals	Mammalia	Class	Mammalia	1258
Conifers	Pinopsida	Class	Pinopsida	198
Crocodiles and alligators	Crocodylia	Order	Reptilia	9
Birches	Betulaceae	Family	Magnoliopsida	19
Magnolias	Magnoliaceae	Family	Magnoliopsida	148
Southern beeches	Nothofagaceae	Family	Magnoliopsida	11
Teas	Theaceae	Family	Magnoliopsida	88
Cacti	Cactaceae	Family	Magnoliopsida	415
Freshwater crabs	Potamidae	Family	Malacostraca	1
Freshwater crabs	Potamonautidae	Family	Malacostraca	2
Freshwater crabs	Gecarcinucidae	Family	Malacostraca	32
Freshwater crabs	Pseudothelphusidae	Family	Malacostraca	1
Chameleons	Chamaeleonidae	Family	Reptilia	74

Supplementary Table 2: Results from a binomial generalized linear model (GLM) of the proportion of threatened species with the appropriate conservation intervention in place. Model structure was of the form **"appropriate_intervention ~ taxonomic_class * rl_category"**, where 'appropriate_intervention' was a binary variable denoting whether the appropriate intervention was in place for each species, as a function of taxonomic class ('taxonomic_class') and Red List Category ('rl_category'), and their interaction. Statistics derive from Likelihood Ratio Tests, dropping each variable in turn and comparing the reduced model to the full model.

Threat	Term	DF	Deviance	P
Habitat loss	taxonomic_class:rl_category	6	25	P = 0.00039
	taxonomic_class	6	123	P = 4.9e-24
	rl_category	1	0.46	P = 0.5
International trade	taxonomic_class:rl_category	4	5.6	P = 0.23
	taxonomic_class	4	70	P = 1.8e-14
	rl_category	1	0.15	P = 0.7
Invasive species	taxonomic_class:rl_category	3	0.092	P = 0.99
	taxonomic_class	3	228	P = 3.4e-49
	rl_category	1	1.6	P = 0.21

Supplementary Table 3: Results from a binomial GLM of the proportion of threatened species with no documented conservation interventions. Model structure was of the form **"no_interventions ~ taxonomic_class * rl_category"**, where 'no_interventions' was a binary variable denoting whether any conservation interventions were documented for each species, as a function of taxonomic class ('taxonomic_class') and Red List Category ('rl_category'), and their interaction. Statistics derive from Likelihood Ratio Tests, dropping each variable in turn and comparing the reduced model to the full model.

Term	DF	Deviance	P
taxonomic_class:rl_category	7	52	P = 5.1e-09
taxonomic_class	7	1530	P = 0
rl_category	1	19	P = 0.000013

Supplementary Table 4: Results from a binomial GLM of the proportion of threatened endemic species with no documented conservation interventions. Model structure was of the form **"no_interventions ~ country"**, where 'no_interventions' was a binary variable denoting whether any conservation interventions were documented for each endemic species, as a function of the country to which it is endemic. Statistics derive from Likelihood Ratio Tests, dropping each variable in turn and comparing the reduced model to the full model.

Term	DF	Deviance	P
country	71	724	P = 0.000013

Supplementary Table 5: Results from a binomial GLM of the model-predicted probability of endemic species receiving no conservation interventions, by country. Model structure was of the form **"prob_no_interventions ~ endemic_n + gdp"**, where 'prob_no_interventions' ranged between 0 and 1 (0 to 100% probability of endemic species receiving no conservation interventions), as a function of the total number of endemic threatened species in the country, and the country's Gross Domestic Product (GDP). Statistics derive from Likelihood Ratio Tests, dropping each variable in turn and comparing the reduced model to the full model.

Term	DF	Deviance	P
endemic_n	1	0.037	P = 0.74
gdp	1	0.0011	P = 0.95

Supplementary References

Hirsch, T., Secretariat of the Convention on Biological Diversity, 2010. Global biodiversity outlook 3. Secretariat of the Convention on Biological Diversity.

IUCN, 2020. The IUCN Red List of Threatened Species. Version 2019-3. [Online]. IUCN Red List of Threatened Species.

Jetz, W., McGowan, J., Rinnan, D.S., Possingham, H.P., Visconti, P., O'Donnell, B., Londoño-Murcia, M.C., 2022. Include biodiversity representation indicators in area-based conservation targets. Nature Ecology & Evolution 6, 123–126. doi:10.1038/s41559-021-01620-y
