# Supplementary material for: Global shortfalls in documented actions to conserve biodiversity
Source: Nature. 2024 Jun 5;630(8016):387–91. doi: 10.1038/s41586-024-07498-7 (PMC11168922; doi:10.1038/s41586-024-07498-7)
Supplement: Supplementary file 2 — Reporting Summary [file 41586_2024_7498_MOESM2_ESM.pdf]

nature research

Corresponding author(s): Rebecca A. Senior

Last updated by author(s): Mar 17, 2024

Reporting Summary

Nature Research wishes to improve the reproducibility of the work that we publish. This form provides structure for consistency and transparency in reporting. For further information on Nature Research policies, see our Editorial Policies and the Editorial Policy Checklist.

Statistics

For all statistical analyses, confirm that the following items are present in the figure legend, table legend, main text, or Methods section.

n/a	Confirmed	
☐	☒	The exact sample size (*n*) for each experimental group/condition, given as a discrete number and unit of measurement
☒	☐	A statement on whether measurements were taken from distinct samples or whether the same sample was measured repeatedly
☐	☒	The statistical test(s) used AND whether they are one- or two-sided<br>*Only common tests should be described solely by name; describe more complex techniques in the Methods section.*
☐	☒	A description of all covariates tested
☐	☒	A description of any assumptions or corrections, such as tests of normality and adjustment for multiple comparisons
☐	☒	A full description of the statistical parameters including central tendency (e.g. means) or other basic estimates (e.g. regression coefficient) AND variation (e.g. standard deviation) or associated estimates of uncertainty (e.g. confidence intervals)
☐	☒	For null hypothesis testing, the test statistic (e.g. *F*, *t*, *r*) with confidence intervals, effect sizes, degrees of freedom and *P* value noted<br>*Give P values as exact values whenever suitable.*
☒	☐	For Bayesian analysis, information on the choice of priors and Markov chain Monte Carlo settings
☒	☐	For hierarchical and complex designs, identification of the appropriate level for tests and full reporting of outcomes
☒	☐	Estimates of effect sizes (e.g. Cohen's *d*, Pearson's *r*), indicating how they were calculated

Our web collection on statistics for biologists contains articles on many of the points above.

Software and code

Policy information about availability of computer code

Data collection	Most data were downloaded manually. Point occurrence data were downloaded in R from the Global Biodiversity Information Facility (GBIF) using the packages 'rgbif' and 'taxize'. Silhouettes used in the figures were downloaded in R from PhyloPic using the package 'rphylopic'. The R version used was 4.3.2.
Data analysis	All data analysis was in R version 4.3.2. The R packages used were: bookdown, cowplot, DiagrammeR, DiagrammeRsvg, dplyr, egg, geodata ggnewscale, ggplot2, ggtext, grid, gridExtra, kableExtra, knitr, lme4, pdftools, png, purrr, RColorBrewer, reticulate, rgbif, rphylopic, rsvg, scales, sf, taxize, tidyr. The Python version used via the R package 'reticulate' was Python 3.9.11. R code to reproduce the results and manuscript is available on GitHub at https://github.com/rasenior/ConservationActions, and is also packaged with the data on Zenodo (DOI: 10.5281/zenodo.10813823).

For manuscripts utilizing custom algorithms or software that are central to the research but not yet described in published literature, software must be made available to editors and reviewers. We strongly encourage code deposition in a community repository (e.g. GitHub). See the Nature Research guidelines for submitting code & software for further information.

Data

Policy information about availability of data

All manuscripts must include a data availability statement. This statement should provide the following information, where applicable:

- Accession codes, unique identifiers, or web links for publicly available datasets
- A list of figures that have associated raw data
- A description of any restrictions on data availability

Processed data to support the findings presented here are available as supplementary data files S1-S3. R code to reproduce the results and figures is available on GitHub at https://github.com/rasenior/ConservationActions, and is also packaged with the data on Zenodo (DOI: 10.5281/zenodo.10813823). The original source

datasets are available for download by request from their respective providers, as described below.

Species data:
• Species assessments can be accessed from the IUCN Red List of Threatened Species website at https://www.iucnredlist.org
• Status change tables are available in pdf format here (Table 7): https://www.iucnredlist.org/resources/summary-statistics
• Range maps can be requested at https://www.iucnredlist.org/resources/spatial-data-download
• Species Protection Scores are available from Map of Life (MOL) at https://mol.org/species/
• Elevation preferences for birds available at https://doi.org/10.1038/nature25794
• Point occurrence records for species with zero HSR were downloaded from the Global Biodiversity Information Facility (GBIF) at https://doi.org/10.15468/DL.DVP728
• EDGE data are available at https://www.edgeofexistence.org/edge-lists/

Conservation interventions data:
• Protected Area boundaries can be requested from the World Database on Protected Areas at https://www.unep-wcmc.org/resources-and-data/analysis/main/wdpa
• The Database of Island Invasive Species Eradications is found at http://diise.islandconservation.org/
• International trade control data (CITES, CMS and EU Annexes) is available from Species+ at https://speciesplus.net/

Geographic data:
• Global Administrative Areas available at https://gadm.org/download_country.html
• Global Islands Database available at https://resources.unep-wcmc.org/products/f98e179ec3f448e59dfe9bda248ff4b6
• Elevation was derived from the EarthEnv Digital Elevation Model Version 1, available at https://www.earthenv.org/DEM
• The global terrestrial habitat types map is available at https://zenodo.org/records/4058819
• Country GDP is available at https://data.worldbank.org/indicator/NY.GDP.MKTP.CD?locations=1W

Field-specific reporting

Please select the one below that is the best fit for your research. If you are not sure, read the appropriate sections before making your selection.

☐ Life sciences ☐ Behavioural & social sciences ☒ Ecological, evolutionary & environmental sciences

For a reference copy of the document with all sections, see nature.com/documents/nr-reporting-summary-flat.pdf

Ecological, evolutionary & environmental sciences study design

All studies must disclose on these points even when the disclosure is negative.

Study description	We assessed the conservation interventions that are in place for threatened species on the IUCN Red List. Statistical analyses considered what factors predict the likelihood that appropriate interventions were in place and the likelihood that any interventions were documented, as well as whether the interventions were associated with changes in species' Red List status. Data were compiled from external sources, and no experimental data were used.
Research sample	We focus only on terrestrial or terrestrial and freshwater species (as defined on the Red List) that are classified as threatened (Vulnerable, Endangered or Critically Endangered) and fall within the taxonomic groups that have been comprehensively assessed to at least family level: birds, mammals, amphibians, chameleons, crocodiles and alligators, freshwater crabs, birches, magnolias, southern beeches, teas, cacti, cycads and conifers. This gave a total of 5963 species. Data sources are described above, and in the Data Availability statement.
Sampling strategy	Not applicable - we used all species with sufficient data for inclusion in the study (i.e. data on threat status, threats, conservation interventions and, for the spatial analyses, spatial distribution data)
Data collection	Data were originally collected by external sources (see above), which provide their own description of how data were compiled and which are unique to each data source.
Timing and spatial scale	IUCN Red List data were downloaded on on 14th July 2020, with data based on multiple assessments by the Red List over time since its conception in the 1980s but particularly since the adoption of standardised assessment criteria in 2000. Spatial scale is global.
Data exclusions	We focus on species described above in 'Research sample', because we were interested in species that are at greatest risk of extinction, and which have sufficient data describing their conservation status and interventions to enable us to draw broad conclusions about the patterns of conservation action.
Reproducibility	Data used are freely available to download, and thus anybody can reproduce our main findings by following the methods described in the manuscript. R code to reproduce the results and manuscript is available on GitHub at https://github.com/rasenior/ConservationActions, and is also packaged with the data on Zenodo (DOI: 10.5281/zenodo.10813823).
Randomization	Not relevant - no experimental data were used
Blinding	Not relevant - no experimental data were used

Did the study involve field work? ☐ Yes ☒ No

Reporting for specific materials, systems and methods

We require information from authors about some types of materials, experimental systems and methods used in many studies. Here, indicate whether each material, system or method listed is relevant to your study. If you are not sure if a list item applies to your research, read the appropriate section before selecting a response.

Materials & experimental systems

n/a	Involved in the study
☒	☐ Antibodies
☒	☐ Eukaryotic cell lines
☒	☐ Palaeontology and archaeology
☒	☐ Animals and other organisms
☒	☐ Human research participants
☒	☐ Clinical data
☒	☐ Dual use research of concern

Methods

n/a	Involved in the study
☒	☐ ChIP-seq
☒	☐ Flow cytometry
☒	☐ MRI-based neuroimaging
